# Mapping topological abnormalities in cortical similarity networks to schizophrenia-associated gene expression

**DOI:** 10.1080/19585969.2026.2682773

**Published:** 2026-06-18

**Authors:** Sung Woo Joo, Jungsun Lee

**Affiliations:** Department of Psychiatry, Asan Medical Center, University of Ulsan College of Medicine, Seoul, Republic of Korea

**Keywords:** Schizophrenia, cortical similarity network, network topology, gene expression

## Abstract

**Introduction:**

Disrupted structural connectivity is recognized as a key pathophysiological feature of schizophrenia (SCZ). However, the relationship between cortical similarity network alterations and gene expression remains poorly understood.

**Methods:**

We applied the Morphometric INverse Divergence framework to T1-weighted MRI from 1,216 participants. Cortical similarity networks were constructed, and global and nodal metrics were computed. Case–control comparisons were performed using linear models. Partial least squares (PLS) regression identified genes associated with spatial patterns of network alterations using Allen Human Brain Atlas data.

**Results:**

Among global metrics, only the rich-club coefficient differed between groups, with a negligible effect size. Nodal metrics showed reduced eigenvector centrality and k-coreness centrality in left temporal/insular (somatomotor), lateral occipital (visual), anterior cingulate (salience/ventral attention), and posterior cingulate (default mode) regions. Participation coefficient was widely reduced in SCZ. For each nodal metric, we identified PLS2-positive and PLS2-negative gene sets. Across degree, eigenvector centrality, and k-coreness centrality, PLS2-negative genes were enriched for metal ion transport, linked to manic and nonorganic psychosis, and upregulated in adolescence and early adulthood. PLS2-positive genes were enriched for neuron projection development and learning or memory, but not psychotic disorders.

**Conclusions:**

These findings highlight synaptic and neurodevelopmental mechanisms underlying structural dysconnectivity in SCZ.

## Introduction

Impairments in structural connectivity beyond regional morphometric alterations represent a critical neurobiological hallmark of schizophrenia (SCZ) (Canu et al. 2015). These findings support the conceptualisation of SCZ as a disorder characterised by disrupted large-scale brain network organisation (Friston et al. 2016). Nonetheless, establishing links between structural abnormalities and clinical manifestations remains challenging. Conventional morphometric network analyses often emphasise regional mean values or inter-regional pairwise similarities, potentially missing higher-order network disruptions relevant to the pathophysiology of SCZ. Moreover, many studies have relied on group-level differences without considering the full topological context of brain organisation.

The Morphometric INverse Divergence (MIND) framework was introduced as a novel method for constructing cortical similarity networks (Sebenius et al. 2023). The MIND framework compares the entire distribution of morphometric features between cortical regions. This allows for the detection of nonlinear and distributional differences, while preserving intra-regional heterogeneity. These properties establish MIND as a robust framework for detecting subtle, spatially complex disruptions in brain structure, particularly in neuropsychiatric disorders such as SCZ. Initial studies using the MIND framework have demonstrated its utility in capturing inter-individual variability, lifespan developmental trajectories, and cognitive associations in healthy populations (Seidlitz et al. 2018; Sebenius et al. 2023). However, applications of MIND to clinical populations remain limited. While earlier studies employing MIND or cortical similarity networks in SCZ have advanced structural characterisation, they have yet to fully integrate topological analyses with molecular data to probe disease-relevant mechanisms (Yao et al. 2024; Fan F et al. 2025; Han et al. 2025).

The use of network metrics has emerged as a robust approach to quantify topological features of brain networks (van den Heuvel and Sporns 2013; Sporns 2018). These include global metrics reflecting whole-network integration and organisation, as well as nodal metrics that capture region-specific roles within the network. Applying these metrics enables a more comprehensive understanding of how specific brain regions contribute to network-level alterations. In SCZ, previous studies have reported reduced segregation and integration in structural networks (Gao et al. 2023), characterised by suboptimal network organisation, reduced wiring efficiency, diminished local clustering, and less hierarchical structure (Micheloyannis 2012). However, most of these studies have relied on diffusion tensor imaging or functional MRI, with relatively few employing cortical similarity networks that integrate multiple morphometric features. Additionally, many were limited by small sample sizes, inconsistent parcellation schemes, or failure to link network alterations to biological mechanisms such as gene expression.

We aimed to apply the MIND framework in a large, multi-site sample of individuals with SCZ and healthy controls to 1) characterise global and nodal topological features of MIND networks; 2) identify spatial patterns of network alterations in SCZ; and 3) link these patterns to gene expression profiles, enabling the identification of genes associated with topological alterations. We sought to uncover the network-level and molecular correlates of structural dysconnectivity in SCZ.

## Materials and methods

### Study population

Participants were recruited from multiple publicly available and institutional datasets including the Centre for Biomedical Research Excellence (COBRE); MIND Clinical Imaging Consortium (MCIC); Neuromorphometry by Computer Algorithm Chicago (NMorphCH); University of California, Los Angeles Consortium for Neuropsychiatric Phenomics LA5c Study (UCLA); and two Korean cohorts from Asan Medical Centre (AMC) and Jeonbuk National University Hospital (JBNU). Diagnoses were based on the Diagnostic and Statistical Manual of Mental Disorders (DSM-IV or DSM-5) criteria. Clinical and cognitive assessments, including the Positive and Negative Syndrome Scale (PANSS) and Full-Scale Intelligence Quotient (FSIQ), were available for a subset of participants. Details of each cohort are provided in the Supplementary Material.

All procedures contributing to this work complied with the ethical standards of the relevant national and institutional committees on human experimentation, as well as with the Declaration of Helsinki guidelines (1975) as revised in 2008. The study was approved by the Institutional Review Board of Asan Medical Centre (IRB No. 2021-1128).

### Image acquisition, preprocessing, and construction of the MIND network

Details regarding the MRI scanners and T1-weighted acquisition parameters are provided in Supplementary Table 1. All MRI data underwent rigorous quality inspection, and only participants with data deemed suitable for analysis were included in the final study sample. T1-weighted images were processed using the automated FreeSurfer pipeline (v7.4; http://surfer.nmr.mgh.harvard.edu). Cortical parcellation was performed using the Schaefer 400-parcel atlas (Schaefer et al. 2018). Quality control was performed using the Euler number (Rosen et al. 2018). A total of 57 outliers were excluded, resulting in a sample of 1,216 participants for the final analysis (Supplementary Figure 1). Detailed demographic and clinical characteristics of the study population are provided in Table 1.

**Table 1.** Demographic and clinical characteristics of the study population.

	AMC 1		AMC 2		AMC 4		COBRE		JBNU		MCIC		NMorphCH		UCLA		Total	
Variable^a^	HC	SCZ	HC	SCZ	HC	SCZ	HC	SCZ	HC	SCZ	HC	SCZ	HC	SCZ	HC	SCZ	HC	SCZ
Number of subjects	23	46	53	25	0	50	82	67	188	392	23	27	39	39	117	45	525	691
Age, mean (SD), years	30.0 (5.4)	28.9 (6.4)	33.8 (7.3)	33.3 (7.5)	NA	38.7 (12.1)	39.1 (11.7)	37.8 (13.0)	37.0 (12.2)	36.4 (12.1)	35.0 (12.0)	32.7 (9.5)	32.0 (8.7)	33.0 (6.9)	31.2 (8.7)	36.6 (9.1)	35.0 (10.9)	35.7 (11.4)
Male, *n* (%)	8 (34.8)	17 (37.0)	15 (28.3)	8 (32.0)	NA	26 (52.0)	60 (73.2)	55 (82.1)	103 (54.8)	196 (50.0)	13 (56.5)	20 (74.1)	18 (46.2)	27 (69.2)	60 (51.3)	35 (77.8)	277 (52.8)	384 (55.6)
Illness duration, mean (SD), years		2.3 (4.0)		9.9 (7.6)		15.9 (9.2)		16.0 (12.5)		9.0 (9.7)		9.7 (8.6)		15.1 (7.6)		NA		10.2 (10.0)
PANSS positive, mean (SD)		16.8 (7.6)		NA		14.3 (4.4)		15.3 (5.2)		13.1 (5.6)		21.5 (3.6)		19.8 (6.9)		17.8 (4.7)		14.7 (6.1)
PANSS negative, mean (SD)		16.7 (6.9)		NA		18.5 (6.5)		17.7 (5.4)		11.6 (5.6)		20.5 (3.2)		21.3 (5.3)		16.2 (5.7)		14.2 (6.6)
FSIQ, mean (SD)	119.9 (9.4)	96.2 (15.6)	NA	NA		90.7 (16.5)	111.0 (13.2)	96.7 (17.2)	NA	79.7 (21.1)	NA	NA	NA	NA	NA	NA	113.0 (12.9)	91.1 (18.9)
Daily OLZ equivalent dose, mean (SD)		17.3 (11.2)		NA		19.0 (12.0)		16.3 (13.0)		NA		NA		NA		19.4 (23.2)		17.8 (15.1)

AMC, Asan Medical Centre; COBRE, Centre for Biomedical Research Excellence; JBNU, Jeonbuk National University Hospital; MCIC, MIND Clinical Imaging Consortium; NMorphCH, Neuromorphometry by Computer Algorithm Chicago; UCLA, University of California Los Angeles Consortium for Neuropsychiatric Phenomics LA5c Study; HC, healthy control; SCZ, schizophrenia; PANSS, Positive and Negative Syndrome Scale; FSIQ, full-scale intelligence quotient; OLZ, olanzapine; SD, standard deviation; NA, not available.

^a^ Information on illness duration, PANSS scores, FSIQ, and antipsychotic medications was available for 603, 644, 312, and 195 subjects, respectively.

Structural brain networks were constructed using the MIND framework (Sebenius et al. 2023). Five surface-based morphometric features (cortical thickness, surface area, gray matter volume, mean curvature, and sulcal depth) were extracted and z-standardized within each participant. For each pair of cortical regions, regional feature distributions were compared using symmetric Kullback–Leibler divergence and converted to bounded similarity scores, yielding a fully connected, weighted similarity matrix for each subject. To address site-related variability across cohorts, we applied neuroCombat harmonisation (Fortin et al. 2018). The effects of harmonisation on connectivity values and global and nodal network metrics are shown in Supplementary Figures 2–4.

**Figure 2.**
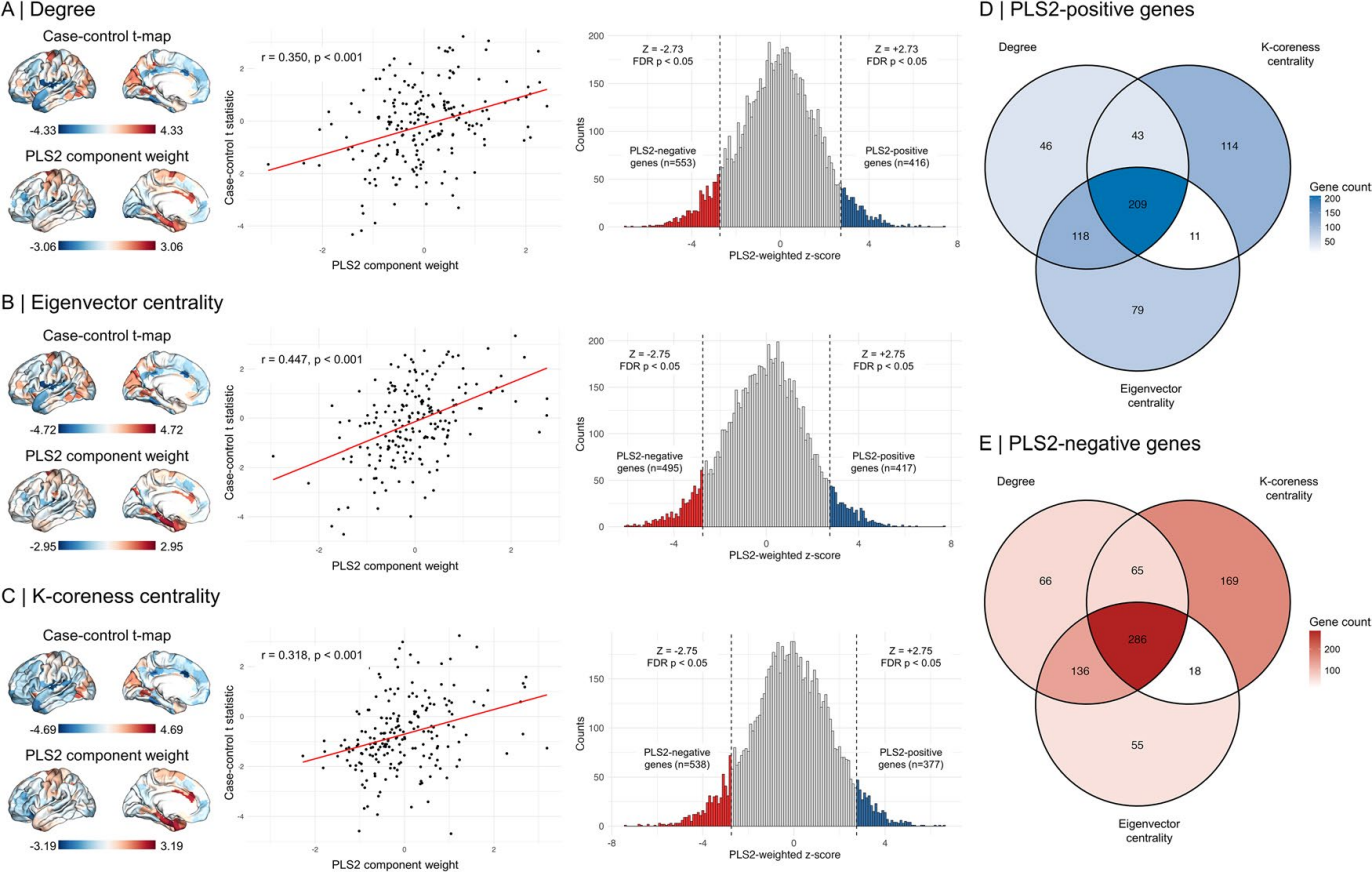
Identification and overlap of PLS2-associated genes across nodal network metrics. (A–C) Correlation between PLS2 component weights and case–control t-statistics for degree (A), eigenvector centrality (B), and k-coreness centrality (C). Scatter plots represent spatial associations across 197 cortical regions. (D–E) Overlap of PLS2-positive (D) and PLS2-negative (E) gene sets identified for each nodal network metric. A substantial proportion of genes were commonly identified across degree, eigenvector centrality, and k-coreness centrality.

**Figure 3.**
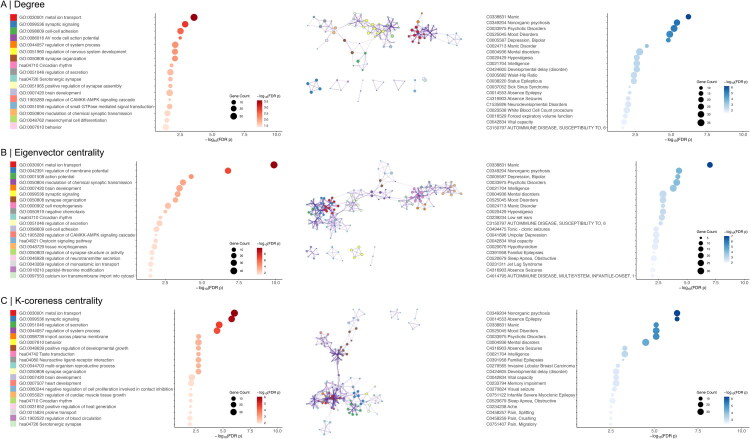
Functional and disease enrichment of PLS2-negative gene sets associated with nodal network metrics. (A–C) Gene Ontology (GO) biological process, KEGG pathway, and disease enrichment results for PLS2-negative gene sets identified from degree (A), eigenvector centrality (B), and k-coreness centrality (C). Dot plots display significantly enriched terms (FDR < 0.05), with colour intensity reflecting –log_10_(p-value) and point size indicating gene count.

### Network metric computation

To determine an appropriate threshold range, small-worldness values were computed across sparsity thresholds ranging from 0.10 to 0.50. For each cohort, small-worldness values were evaluated across thresholds (Supplementary Figure 5). Based on the proportion of valid subjects and the criterion of small-worldness σ > 1.2, the optimal sparsity range was determined to be 0.25–0.50. Network metrics were calculated using the Brain Connectivity Toolbox (Rubinov and Sporns 2010). To comprehensively capture the topological characteristics of the MIND networks, the following global network metrics were computed: global efficiency, transitivity, modularity (Q), maximum rich-club coefficient, and assortativity coefficient. Nodal network metrics included degree, eigenvector centrality, k-coreness centrality, local efficiency, participation coefficient, and betweenness centrality. Detailed descriptions of each metric are provided in the Supplementary Material.

For group comparisons, linear regression models were applied to each metric, controlling for age, sex, and estimated total intracranial volume for global metrics, and for regional gray matter volume for nodal metrics. The results were corrected for multiple comparisons using the Benjamini–Hochberg false discovery rate (FDR) method, considering the number of global metrics (*n* = 5) and number of cortical regions for nodal metrics (*n* = 400), respectively. The 400 cortical regions were grouped into seven functional networks defined by Yeo et al. (Yeo et al. 2011). Differences in t-statistics across networks were assessed using analysis of variance (ANOVA), followed by post-hoc testing with Tukey’s honestly significant difference (HSD) test. To assess the similarity in the distributional patterns of case-control t-statistics across nodal metrics, Pearson correlation analyses were conducted. Exploratory analyses were performed using Spearman’s correlation to evaluate the relationship between nodal network metrics and clinical variables. Correlation analyses with clinical variables were conducted only for nodal network metrics with significant group differences and were based on available clinical data only, without imputation for missing values. Results were corrected for multiple comparisons using the FDR method within each clinical variable.

### Linking network metrics to gene expression

We used microarray expression data from six adult human donor brains provided by the Allen Human Brain Atlas (AHBA) (Hawrylycz et al. 2012). Preprocessing was performed using the abagen toolbox (Arnatkeviciute et al. 2019). Genes with cross-donor expression similarity above a correlation threshold (*r* ≥ 0.2) were retained, resulting in a total of 7,584 genes for analysis. Owing to incomplete right-hemisphere sampling in two donors, all transcriptomic analyses were restricted to the left hemisphere. A gene expression matrix comprising 197 cortical regions and 7,584 genes was used for subsequent analyses, excluding three regions with missing gene expression data.

To investigate the association between spatial pattern of topological alterations and gene expression, we performed partial least squares (PLS) regression (Abdi 2010; Arnatkeviciute et al. 2019). The dependent variable was a vector of z-normalised case-control t-statistics for each nodal network metric across 197 regions. The predictor matrix consisted of normalised expression values of the retained genes. The number of PLS components was determined using 5,000-permutation testing, in which the variance explained by each component was compared against a null distribution generated by randomly permuting the response variable. Statistical analyses were performed using R software (version 4.2.1) with pls package. To identify genes significantly contributing to the selected PLS component, we conducted bootstrap resampling (10,000 iterations) across brain regions. For each gene, a z-score was calculated by dividing its weight on the selected component by the standard error derived from the bootstrap distribution. Genes with FDR-corrected *p*-values below 0.05 were considered significant and classified as PLS-positive or PLS-negative based on the sign of their respective weights.

### Biological characterisation of PLS-associated genes

To interpret the biological significance of PLS-associated genes, we conducted functional enrichment, developmental trajectory analysis, and disease association profiling. Functional annotation was performed using Metascape (https://metascape.org) (Zhou et al. 2019), focusing on Gene Ontology (GO) biological processes and Kyoto Encyclopaedia of Genes and Genomes (KEGG) pathways. Enrichment was assessed using a hypergeometric test with FDR correction (*q* < 0.05), and redundant terms were grouped into functional clusters using Kappa statistics (similarity threshold = 0.3), with a maximum of 15 terms per cluster. Developmental expression profiles were examined using cortical transcriptomic data from the PsychENCODE developmental atlas (Li M et al. 2018). Expression values were log-transformed, mean-centered, and averaged across cortical regions within each window. To assess developmental differences, we applied a linear mixed-effects model with the developmental period as a fixed effect and cortical region as a random effect. Disease relevance was assessed using enrichment analysis *via* DisGeNET (Piñero et al. 2017). Fisher’s exact test was used to evaluate the overrepresentation of disease-associated genes within each PLS gene set, with significance defined as an FDR-corrected *p* < 0.05.

## Results

### Group comparisons of global and nodal network metrics

Supplementary Table 2 summarises the group comparisons of global network metrics. No significant differences were observed in the global efficiency, transitivity, modularity, or assortativity coefficient. Although the rich-club coefficient was significantly higher in the SCZ group (FDR *p* = 0.007), the absolute difference was negligible.

**Figure 1.**
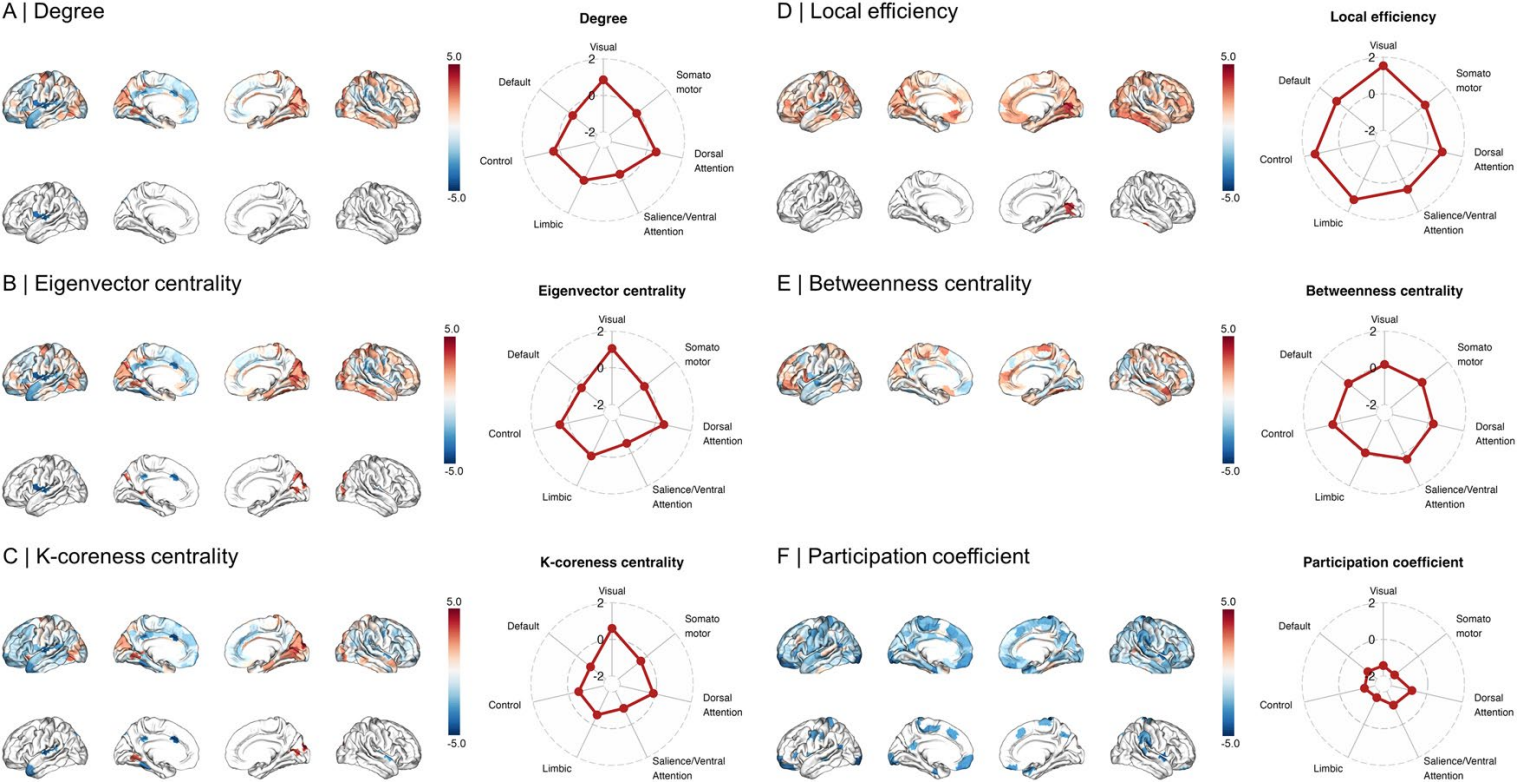
Group comparisons of nodal network metrics between individuals with schizophrenia and healthy controls. (A–F) Brain surface maps display vertex-wise case-control t-statistics for each nodal network metric: (A) degree, (B) eigenvector centrality, (C) k-coreness centrality, (D) local efficiency, (E) betweenness centrality, and (F) participation coefficient. Regions with FDR-corrected *p*-values < 0.05 are indicated below each corresponding surface map. Warm colours indicate increased t-statistic in SCZ relative to HC, whereas cool colours represent decreased t-statistic in SCZ relative to HC. Radar plots to the right of each map summarise the mean t-statistics across the seven Yeo functional networks.

Figure 1 presents the results of nodal network metric comparisons between the groups. In individuals with SCZ, reductions in degree, eigenvector centrality, and k-coreness centrality were commonly observed in the left temporal and insular areas within the somatomotor network, as well as in the left lateral occipital area within the visual network, compared to healthy controls. Eigenvector centrality and k-coreness centrality were also consistently reduced in the left posterior cingulate area (default mode network), the left anterior cingulate area (salience/ventral attention network), Heschl’s gyrus (somatomotor network), and the left temporal area (visual network) in individuals with SCZ. In contrast, increased values for both nodal network metrics were observed in individuals with SCZ in two right occipital areas within the visual network and the right postcentral area within the somatomotor network. A reduction in k-coreness centrality was found in the left temporal pole (limbic network), while a significant increase in local efficiency was observed in the right temporal pole. Additionally, local efficiency was significantly increased in two right occipital areas within the visual network in individuals with SCZ.

Supplementary Figure 6 shows the Pearson correlation matrix of case-control t-statistics across nodal network metrics. Degree, eigenvector centrality, and k-coreness centrality showed similar spatial distribution patterns (*r* > 0.7). Participation coefficient demonstrated a markedly different pattern. Supplementary Table 3 and Supplementary Figure 7-8 illustrate case-control t-statistics of nodal network metrics aggregated by the Yeo functional network. The visual network showed a general tendency towards higher t-statistics, while the default network consistently exhibited lower values than other nodal network metrics, a trend most evident for k-coreness centrality.

Supplementary Table 4 presents the significant correlations between the nodal network metrics and clinical variables. A number of significant associations were observed between the participation coefficient and PANSS negative subscale scores and FSIQ. However, most of these correlations had rho values below 0.3, indicating weak associations.

### Selection of PLS2-associated gene sets

Supplementary Figure 9 shows the results of permutation tests used to determine the optimal number of PLS components. A two-component PLS model was identified as appropriate for degree, eigenvector centrality, k-coreness centrality, and local efficiency. Across these metrics, the variance explained by the PLS2 component exceeded that of PLS1 (degree: 7.27% vs. 14.38%; eigenvector centrality: 6.41% vs. 20.82%; k-coreness centrality: 11.23% vs. 13.06%; local efficiency: 5.75% vs. 20.21%), and thus, the PLS2 component was used to identify the associated gene sets.

Genes were classified as PLS2-associated-positive or PLS2-associated-negative based on their directionality of association with the nodal network metric. The PLS2-positive gene set comprises genes highly expressed in regions showing increased nodal metric values in SCZ, whereas the PLS2-negative gene set includes genes enriched in regions with reduced nodal metric values. No genes showed significant associations with local efficiency. Figure 2A–C illustrates the correlations between PLS2 component weights and case–control t-statistics for degree, eigenvector centrality, and k-coreness centrality. Figure 2D–E shows the overlap between PLS2-positive and PLS2-negative genes associated with degree, eigenvector centrality, and k-coreness centrality. A substantial proportion of the genes identified for each nodal network metric were shared across all three metrics.

### Functional, developmental, disease association with PLS2-positive and negative gene sets

Figure 3 presents the GO and KEGG pathway enrichment results for the PLS2-negative gene sets associated with degree, eigenvector centrality, and k-coreness centrality. Among all three nodal network metrics, the most prominent enrichment was observed for metal ion transport. Disease enrichment analysis revealed significant associations with manic and nonorganic psychosis across all three metrics. Supplementary Figure 10 presents the GO and KEGG pathway enrichment results for the PLS2-positive gene sets. While enrichment patterns varied across nodal network metrics, all three showed significant associations with terms related to learning or memory and neuron projection development. No significant disease associations were identified for k-coreness centrality, whereas gene sets associated with degree and eigenvector centrality commonly showed enrichment for childhood astrocytoma.

To investigate developmental associations, we examined the expression profiles of PLS2-associated gene sets using the PsychENCODE developmental atlas. Significant group × window interactions were observed across all three nodal network metrics (degree: *F* = 4.138, *p* < 0.001; eigenvector centrality: *F* = 7.094, *p* < 0.001; k-coreness centrality: *F* = 7.090, *p* < 0.001) (Supplementary Figure 11). Post hoc analysis showed that PLS2-negative genes had significantly higher expression than PLS2-positive genes during adolescence and early adulthood for eigenvector and k-coreness centrality, and during early adulthood only for degree (Supplementary Table 5).

## Discussion

We constructed cortical similarity networks using the MIND framework in a large sample. Most global metrics showed no significant group differences. In contrast, nodal-level alterations were evident, with degree, eigenvector centrality, and k-coreness centrality showing similar spatial patterns. The participation coefficient showed a distinct pattern with widespread reductions in SCZ. PLS2-negative gene sets for degree, eigenvector centrality, and k-coreness centrality were enriched for metal ion transport pathways and associated with manic and nonorganic psychosis, whereas PLS2-positive gene sets were enriched for neuron projection development and learning or memory pathways without psychosis associations.

Although the rich-club coefficient differed between groups, the effect size was small, indicating preserved hub backbone integrity. In line with our earlier work (Joo et al. 2024), the current results indicate that global network organisation remains largely intact in SCZ. In contrast, Gao et al. reported reductions in segregation and integration in the structural connectome of SCZ, though subject to clinical and methodological variability across individual studies (Gao et al. 2023). Individuals with SCZ exhibited reduced degree, eigenvector centrality, and k-coreness centrality in the left temporal and insular regions associated with the somatomotor network, as well as with the lateral occipital region of the visual network. These results align with prior reports of decreased intra-network structural and/or functional connectivity within the somatomotor and visual networks in SCZ (Reavis et al. 2020; Fan L et al. 2022; Keyvanfard et al. 2023). Eigenvector and k-coreness centrality were reduced in individuals with SCZ in the left anterior cingulate (salience/ventral attention network) and left posterior cingulate (default mode network). As both metrics reflect nodal importance, their reductions suggest disrupted hub integrity in these regions, consistent with prior reports of functional abnormalities in these networks in schizophrenia (White et al. 2010; Palaniyappan et al. 2012; Hu et al. 2017). The spatial distribution of participation coefficient t-statistics differed from other nodal metrics. Given that the participation coefficient reflects cross-network integration, widespread reductions in SCZ suggest disrupted inter-network connectivity (Orliac et al. 2017; Ishida et al. 2023; Metzner et al. 2024). Local efficiency showed a general increase in SCZ, with several region-specific elevations. Increased local efficiency may reflect compensatory responses to disrupted long-range or inter-network connectivity (Cai et al. 2022; Wei et al. 2022). Several nodes with reduced participation coefficient showed weak but statistically significant correlations with the PANSS negative subscale score and FSIQ. However, these exploratory findings warrant cautious interpretation. Alterations in intrinsic network topology may reflect higher-order organisational constraints shaping clinical expression, consistent with prior work linking large-scale brain organisation to cognition and behaviour despite modest symptom correlations (Mo et al. 2024; Zhang et al. 2024; Cheng et al. 2025; Li Y et al. 2025). Multivariate and longitudinal approaches may yield more robust clinical insights.

We used PLS regression to identify genes associated with regional MIND network topology in SCZ. As disease-related alterations may deviate from dominant global covariance, we focused on secondary PLS components capturing potentially disease-relevant residual variation. PLS2-positive and negative gene sets were identified and examined for functional, developmental, and disease associations. This approach aligns with prior neuroimaging–transcriptome studies linking brain-wide patterns to gene expression in SCZ (Li Q et al. 2023; Xu et al. 2023). The PLS2-negative genes were associated with pathways related to metal ion transport and synaptic signalling. Several SCZ-associated genes are involved in the regulation of essential metal ions across neuronal membranes (Ripke et al. 2014; Pers et al. 2016). Genetic studies have identified polymorphisms in metal ion transporter genes as risk factors for SCZ (Mealer et al. 2020). Synaptic dysfunction has emerged as a key mechanism in the pathophysiology of SCZ. Genetic and transcriptomic studies consistently implicate genes involved in synapse organisation and trans-synaptic signalling in increased susceptibility to the disorder (Lips et al. 2012; Fromer et al. 2014; Purcell et al. 2014). Disease enrichment analysis showed associations with manic and nonorganic psychosis, suggesting that PLS2-negative genes may reflect a psychosis-related risk profile. Developmentally, PLS2-negative gene expression was elevated during adolescence and early adulthood, consistent with neurodevelopmental models of SCZ (Howes and Onwordi 2023). The PLS2-positive genes were enriched for pathways related to neuron projection development and learning or memory. Given prior evidence implicating neuronal development (Jaaro-Peled et al. 2009) and cognitive dysfunction (McCutcheon et al. 2023) in SCZ, the PLS2-positive genes may have functional relevance. However, their disease associations were unrelated to psychosis, suggesting that these genes may reflect adaptive or compensatory processes, or broader neurodevelopmental mechanisms rather than core psychosis pathology. The biological implications of these increased morphometric features remain unclear.

Compared with previous studies examining cortical similarity networks and/or transcriptional profiles in psychosis (Morgan et al. 2019; Yao et al. 2023; Yao et al. 2024; Fan F et al. 2025; Han et al. 2025), the present study provides several methodological and interpretative advantages. First, the larger sample size improves representativeness and sensitivity to subtle group differences. Second, we applied multiple network metrics to comprehensively characterise node-level abnormalities in cortical similarity networks in SCZ. Third, the MIND framework enables the detection of nonlinear and distributional differences with enhanced sensitivity and granularity in topological analyses (Sebenius et al. 2023). However, several limitations should be considered. First, we applied ComBat harmonisation to minimise site-related variability; however, residual site effects may remain. Second, the AHBA includes data from only six healthy donors, and gene expression information is restricted to the left hemisphere. Accordingly, associations between network topology and gene expression should be interpreted with caution due to hemispheric restriction and require validation using bilateral transcriptomic data. Third, clinical information was not available for all participants, which limited multivariate adjustment for potential confounders, rendering associations between nodal network metrics and clinical variables exploratory and warranting cautious interpretation. Fourth, the cross-sectional design precludes conclusions about causal relationships. Fifth, this multi-site cohort included several sites with relatively small sample sizes, and heterogeneity in participant characteristics across sites may have influenced the results.

In conclusion, this study leveraged the MIND framework to construct cortical similarity networks in a large multi-site sample. Using global and nodal network metrics, we identified region-specific topological alterations in SCZ, particularly in the somatomotor, visual, salience/ventral attention, and default mode networks. We linked these network-level disruptions to distinct gene sets *via* PLS regression. The PLS2-positive gene set was enriched for neuron projection development and learning or memory pathways, but showed no associations with psychosis. The PLS2-negative gene set was associated with metal ion transport and synaptic signalling and showed disease associations potentially specific to SCZ. Future studies integrating developmental transcriptomics and richer phenotypic data are warranted to further elucidate the molecular and network-level mechanisms underlying SCZ.

## Supplementary Material

Supplementary_File_v2_clean.docx

## References

[CIT0001] Abdi H. 2010. Partial least squares regression and projection on latent structure regression (PLS Regression). WIREs Computational Stats. 2(1):97–106. 10.1002/wics.51

[CIT0002] Arnatkeviciute A, Fulcher BD, Fornito A. 2019. A practical guide to linking brain-wide gene expression and neuroimaging data. Neuroimage. 189:353–367. 10.1016/j.neuroimage.2019.01.011 30648605

[CIT0003] Cai M et al. 2022. Disrupted local functional connectivity in schizophrenia: an updated and extended meta-analysis. Schizophrenia (Heidelb). 8(1):93. 10.1038/s41537-022-00311-2 36347874 PMC9643538

[CIT0004] Canu E, Agosta F, Filippi M. 2015. A selective review of structural connectivity abnormalities of schizophrenic patients at different stages of the disease. Schizophr Res. 161(1):19–28. 10.1016/j.schres.2014.05.020 24893909

[CIT0005] Cheng Y et al. 2025. Brain network localization of gray matter atrophy and neurocognitive and social cognitive dysfunction in schizophrenia. Biol Psychiatry. 97(2):148–156. 10.1016/j.biopsych.2024.07.021 39103010

[CIT0006] Fan F et al. 2025. Aberrant cortical morphological networks in first-episode schizophrenia. Schizophr Bull. 51(5):1351–1366. 10.1093/schbul/sbae218 39761216 PMC12414567

[CIT0007] Fan L et al. 2022. Aberrant large-scale brain modules in deficit and non-deficit schizophrenia. Prog Neuropsychopharmacol Biol Psychiatry. 113:110461. 10.1016/j.pnpbp.2021.110461 34688810

[CIT0008] Fortin J-P et al. 2018. Harmonization of cortical thickness measurements across scanners and sites. Neuroimage. 167:104–120. 10.1016/j.neuroimage.2017.11.024 29155184 PMC5845848

[CIT0009] Friston K, Brown HR, Siemerkus J, Stephan KE. 2016. The dysconnection hypothesis (2016). Schizophr Res. 176(2-3):83–94. 10.1016/j.schres.2016.07.014 27450778 PMC5147460

[CIT0010] Fromer M et al. 2014. De novo mutations in schizophrenia implicate synaptic networks. Nature. 506(7487):179–184. 10.1038/nature12929 24463507 PMC4237002

[CIT0011] Gao Z et al. 2023. The whole-brain connectome landscape in patients with schizophrenia: a systematic review and meta-analysis of graph theoretical characteristics. Neurosci Biobehav Rev. 148:105144. 10.1016/j.neubiorev.2023.105144 36990373

[CIT0012] Han Y et al. 2025. Cortical morphometric similarity gradient in schizophrenia and its association with transcriptional profiles and clinical phenotype. Psychol Med. 55:e97. 10.1017/S0033291725000479 40143806 PMC12094636

[CIT0013] Hawrylycz MJ et al. 2012. An anatomically comprehensive atlas of the adult human brain transcriptome. Nature. 489(7416):391–399. 10.1038/nature11405 22996553 PMC4243026

[CIT0014] Howes OD, Onwordi EC. 2023. The synaptic hypothesis of schizophrenia version III: a master mechanism. Mol Psychiatry. 28(5):1843–1856. 10.1038/s41380-023-02043-w 37041418 PMC10575788

[CIT0015] Hu ML et al. 2017. A review of the functional and anatomical default mode network in schizophrenia. Neurosci Bull. 33(1):73–84. 10.1007/s12264-016-0090-1 27995564 PMC5567552

[CIT0016] Ishida T et al. 2023. Aberrant large-scale network interactions across psychiatric disorders revealed by large-sample multi-site resting-state functional magnetic resonance imaging datasets. Schizophr Bull. 49(4):933–943. 10.1093/schbul/sbad022 36919870 PMC10318885

[CIT0017] Jaaro-Peled H et al. 2009. Neurodevelopmental mechanisms of schizophrenia: understanding disturbed postnatal brain maturation through neuregulin-1-ErbB4 and DISC1. Trends Neurosci. 32(9):485–495. 10.1016/j.tins.2009.05.007 19712980 PMC2755075

[CIT0018] Joo SW et al. 2024. Topological abnormalities of the morphometric similarity network of the cerebral cortex in schizophrenia. Schizophrenia (Heidelb). 10(1):57. 10.1038/s41537-024-00477-x 38886369 PMC11183129

[CIT0019] Keyvanfard F, Schmid AK, Nasiraei-Moghaddam A. 2023. Functional connectivity alterations of within and between networks in schizophrenia: a retrospective study. Basic Clin Neurosci. 14(3):397–409. 10.32598/bcn.2022.3928.2 38077180 PMC10700811

[CIT0020] Li M et al. 2018. Integrative functional genomic analysis of human brain development and neuropsychiatric risks. Science. 362(6420):eaat7615. 10.1126/science.aat7615 PMC6413317 30545854

[CIT0021] Li Q et al. 2023. Resting-state brain functional alterations and their genetic mechanisms in drug-naive first-episode psychosis. Schizophrenia (Heidelb). 9(1):13. 10.1038/s41537-023-00338-z 36841861 PMC9968350

[CIT0022] Li Y et al. 2025. Brain structural and functional alteration network localization of visual hallucinations. Schizophr Bull. sbaf162. 10.1093/schbul/sbaf162 PMC13391636 40973154

[CIT0023] Lips ES et al. 2012. Functional gene group analysis identifies synaptic gene groups as risk factor for schizophrenia. Mol Psychiatry. 17(10):996–1006. 10.1038/mp.2011.117 21931320 PMC3449234

[CIT0024] McCutcheon RA, Keefe RSE, McGuire PK. 2023. Cognitive impairment in schizophrenia: aetiology, pathophysiology, and treatment. Mol Psychiatry. 28(5):1902–1918. 10.1038/s41380-023-01949-9 36690793 PMC10575791

[CIT0025] Mealer RG et al. 2020. The schizophrenia risk locus in SLC39A8 alters brain metal transport and plasma glycosylation. Sci Rep. 10(1):13162. 10.1038/s41598-020-70108-9 32753748 PMC7403432

[CIT0026] Metzner C et al. 2024. Exploring global and local processes underlying alterations in resting-state functional connectivity and dynamics in schizophrenia. Front Psychiatry. 15:1352641. 10.3389/fpsyt.2024.1352641 38414495 PMC10897003

[CIT0027] Micheloyannis S. 2012. Graph-based network analysis in schizophrenia. World J Psychiatry. 2(1):1–12. 10.5498/wjp.v2.i1.1 24175163 PMC3782171

[CIT0028] Mo F et al. 2024. Network localization of state and trait of auditory verbal hallucinations in schizophrenia. Schizophr Bull. 50(6):1326–1336. 10.1093/schbul/sbae020 38401526 PMC11548935

[CIT0029] Morgan SE et al. 2019. Cortical patterning of abnormal morphometric similarity in psychosis is associated with brain expression of schizophrenia-related genes. Proc Natl Acad Sci U S A. 116(19):9604–9609. 10.1073/pnas.1820754116 31004051 PMC6511038

[CIT0030] Orliac F et al. 2017. Network modeling of resting state connectivity points towards the bottom up theories of schizophrenia. Psychiatry Res Neuroimaging. 266:19–26. 10.1016/j.pscychresns.2017.04.003 28554165

[CIT0031] Palaniyappan L, White TP, Liddle PF. 2012. The concept of salience network dysfunction in schizophrenia: from neuroimaging observations to therapeutic opportunities. Curr Top Med Chem. 12(21):2324–2338. 10.2174/156802612805289881 23279173

[CIT0032] Pers TH, Consortium SWGotPG. et al. 2016. Comprehensive analysis of schizophrenia-associated loci highlights ion channel pathways and biologically plausible candidate causal genes. Hum Mol Genet. 25(6):1247–1254. 10.1093/hmg/ddw007 26755824 PMC4764200

[CIT0033] Piñero J et al. 2017. DisGeNET: a comprehensive platform integrating information on human disease-associated genes and variants. Nucleic Acids Res. 45(D1):D833–D839. 10.1093/nar/gkw943 27924018 PMC5210640

[CIT0034] Purcell SM et al. 2014. A polygenic burden of rare disruptive mutations in schizophrenia. Nature. 506(7487):185–190. 10.1038/nature12975 24463508 PMC4136494

[CIT0035] Reavis EA et al. 2020. Structural and functional connectivity of visual cortex in schizophrenia and bipolar disorder: a graph-theoretic analysis. Schizophr Bull Open. 1(1):sgaa056. 10.1093/schizbullopen/sgaa056 33313506 PMC7712743

[CIT0036] Ripke S et al. 2014. Biological insights from 108 schizophrenia-associated genetic loci. Nature. 511(7510):421–427. 25056061 10.1038/nature13595 PMC4112379

[CIT0037] Rosen AFG et al. 2018. Quantitative assessment of structural image quality. Neuroimage. 169:407–418. 10.1016/j.neuroimage.2017.12.059 29278774 PMC5856621

[CIT0038] Rubinov M, Sporns O. 2010. Complex network measures of brain connectivity: uses and interpretations. Neuroimage. 52(3):1059–1069. 10.1016/j.neuroimage.2009.10.003 19819337

[CIT0039] Schaefer A et al. 2018. Local-global parcellation of the human cerebral cortex from intrinsic functional connectivity MRI. Cereb Cortex. 28(9):3095–3114. eng. 10.1093/cercor/bhx179 28981612 PMC6095216

[CIT0040] Sebenius I et al. 2023. Robust estimation of cortical similarity networks from brain MRI. Nat Neurosci. 26(8):1461–1471. 10.1038/s41593-023-01376-7 37460809 PMC10400419

[CIT0041] Seidlitz J et al. 2018. Morphometric similarity networks detect microscale cortical organization and predict inter-individual cognitive variation. Neuron. 97(1):231–247.e7. e237. eng. 10.1016/j.neuron.2017.11.039 29276055 PMC5763517

[CIT0042] Sporns O. 2018. Graph theory methods: applications in brain networks. Dialogues Clin Neurosci. 20(2):111–121. 10.31887/DCNS.2018.20.2/osporns 30250388 PMC6136126

[CIT0043] van den Heuvel MP, Sporns O. 2013. Network hubs in the human brain. Trends Cogn Sci. 17(12):683–696. 10.1016/j.tics.2013.09.012 24231140

[CIT0044] Wei J et al. 2022. Functional integration and segregation in a multilayer network model of patients with schizophrenia. Brain Sci. 12(3):368. 10.3390/brainsci12030368 35326324 PMC8946586

[CIT0045] White TP, Joseph V, Francis ST, Liddle PF. 2010. Aberrant salience network (bilateral insula and anterior cingulate cortex) connectivity during information processing in schizophrenia. Schizophr Res. 123(2-3):105–115. 10.1016/j.schres.2010.07.020 20724114

[CIT0046] Xu X et al. 2023. Genetic mechanisms underlying gray matter volume changes in patients with drug-naive first-episode schizophrenia. Cereb Cortex. 33(5):2328–2341. 10.1093/cercor/bhac211 35640648

[CIT0047] Yao G et al. 2024. Transcriptional patterns of the cortical Morphometric Inverse Divergence in first-episode, treatment-naive early-onset schizophrenia. Neuroimage. 285:120493. 10.1016/j.neuroimage.2023.120493 38086496

[CIT0048] Yao G et al. 2023. Cortical structural changes of morphometric similarity network in early-onset schizophrenia correlate with specific transcriptional expression patterns. BMC Med. 21(1):479. 10.1186/s12916-023-03201-1 38049797 PMC10696871

[CIT0049] Yeo BTT et al. 2011. The organization of the human cerebral cortex estimated by intrinsic functional connectivity. J Neurophysiol. 106(3):1125–1165. 10.1152/jn.00338.2011 21653723 PMC3174820

[CIT0050] Zhang X, Xu R, Ma H, Qian Y, Zhu J. 2024. Brain structural and functional damage network localization of suicide. Biol Psychiatry. 95(12):1091–1099. 10.1016/j.biopsych.2024.01.003 38215816

[CIT0051] Zhou Y et al. 2019. Metascape provides a biologist-oriented resource for the analysis of systems-level datasets. Nat Commun. 10(1):1523. 10.1038/s41467-019-09234-6 30944313 PMC6447622

