# Supplementary material for: Mapping topological abnormalities in cortical similarity networks to schizophrenia-associated gene expression
Source: Dialogues Clin Neurosci. 2026 Jun 18;28(1):240–9. doi: 10.1080/19585969.2026.2682773 (PMC13288719; doi:10.1080/19585969.2026.2682773)
Supplement: Supplementary_File_v2_clean.docx [file TDCN_A_2682773_SM2469.docx]

**Supplementary Material**

**Study population**

We included a total of 1,216 participants in the analysis; the cohort consisted of 691 patients with schizophrenia and 525 healthy controls. Participants were drawn from multiple publicly available and institutional magnetic resonance imaging (MRI) datasets, including the Center for Biomedical Research Excellence (COBRE), Mind Clinical Imaging Consortium (MCIC), Neuromorphometry by computer algorithm Chicago (NMorphCH), University of California Los Angeles Consortium for Neuropsychiatric Phenomics LA5c Study (UCLA), and two Korean cohorts from Asan Medical Center (AMC) and Jeonbuk National University Hospital (JBNU). The details of each cohort are described below.

**AMC**

Participants were included from Asan Medical Center, a university-affiliated hospital. The cohorts (AMC 1, AMC 2, and AMC 3) were designed to investigate neurobiological alterations in schizophrenia or bipolar disorder. Chronologically, we began with the inclusion of the AMC 1 cohort, followed by AMC 2, and then AMC 3. In each cohort, psychiatric symptoms and cognitive functions were assessed using different tools. Below, we describe the specifics of each cohort, including the inclusion and exclusion criteria, as well as clinical information.

1. **AMC 1**

An experienced psychiatrist diagnosed patients according to the Diagnostic and Statistical Manual of Mental Disorders-IV (DSM-IV) criteria. For all patients, the onset of the first psychotic symptoms, including delusion or hallucination, occurred five or less than five years before enrollment. Subjects in the control group and their first-degree relatives had no Axis I psychiatric diagnoses based on the DSM-IV-TR criteria. All the subjects were right-handed and aged 20–40 years. Exclusion criteria were as follows: diseases that affect brain function, or inability to complete neuropsychological testing or the MRI scanning session. We excluded 20 subjects owing to poor image quality, incidental brain lesions, or a change in the original diagnosis to that of other psychotic disorders (e.g., bipolar disorder) when we re-evaluated them 1–6 months after enrollment. All the subjects provided written informed consent before enrollment, and ethical approval for the study was obtained from the Institutional Review Board of Asan Medical Center (IRB File No. 2012-0485).

Assessment of symptoms, neurocognition, and social cognition was completed within a week from the date of the MRI scan. All subjects were evaluated by the age- and sex-adjusted short form of the Wechsler Adult Intelligence Scale-Third edition (WAIS-III), and the video-based social cognition scale (VISC). The age- and sex-adjusted short form of the WAIS consists of six subtests, including the digit span, vocabulary, arithmetic, picture arrangement, block design, and digit symbol tests. The VISC is a scale for evaluating social cognition based on video clips. The scale consists of 20 video clips portraying frequently experienced social interactions in real life. Patients were asked to point out socially unnatural behavior or speech, or to state the reasons for lies made by actors or actresses. Each question had a scoring scale ranging from 0–2, with a maximum total score of 40. The psychiatric symptoms of the patients were rated using the Positive and Negative Syndrome Scale (PANSS).

1. **AMC 2**

Participants were recruited between August 2017 and February 2020. The patient group included individuals diagnosed with schizophrenia or bipolar disorder. One of the main objectives of this cohort was to investigate structural and functional similarities and differences between schizophrenia and bipolar disorder. Diagnoses in the patient group were made using the Structured Clinical Interview for DSM Disorders (SCID). The control group comprised participants who met the following criteria: (1) no history of Axis I diagnosis according to the SCID and (2) no history of Axis I diagnosis in first-degree relatives according to the SCID. Additional inclusion criteria for both groups were as follows: (1) age between 20 and 55 years, (2) right-handedness, (3) Full-Scale Intelligence Quotient (FSIQ) > 80, and (4) absence of any physical illness that could interfere with brain function. After visual inspection of the MRI data, the final sample included 27 patients with schizophrenia, 24 with bipolar disorder, and 55 healthy controls. Written informed consent was obtained from all the participants before enrollment, and the study received ethical approval from the Institutional Review Board of Asan Medical Center (IRB File No. 2017-0839).

Neurocognitive function was assessed using the Cogstate Brief Battery, which evaluates the following seven cognitive domains: processing speed, attention, working memory, visual learning and memory, verbal learning and memory, executive functions, and social cognition.

1. **AMC 3**

Participants were recruited between June 2021 and December 2023. The inclusion criteria were as follows: (1) diagnosis of schizophrenia according to the Diagnostic and Statistical Manual of Mental Disorders 5 (DSM-5) criteria; (2) illness duration > 5 years; and 3) right-handedness. Participants were excluded based on the following criteria: (1) history of intellectual disability; (2) history of substance abuse or dependency in the last six months; (3) history of head trauma leading to over 3 min of unconsciousness; (4) neurological disorders; and (5) unstable diseases that may interfere with brain function. All participants provided written informed consent before enrollment. A total of 69 patients with schizophrenia were enrolled, with 14 withdrawing consent, resulting in 55 patients who completed the study protocol. This study received approval from the Institutional Review Board (IRB) of the Asan Medical Center (AMC) (IRB File Nos. 2021-1128, 2022-1193) and was conducted according to the Declaration of Helsinki guidelines. The final study population consisted of 52 patients with schizophrenia, after exclusion of three patients owing to poor image quality (n = 2) and major neurological malformation (n = 1).

Interviews and evaluations of the participants were conducted one week before the MRI scan. Clinical information, including histories of previous and current illness, family, and antipsychotic treatment, was obtained. The severity of psychiatric symptoms was assessed using the PANSS. Cognitive functions were measured using a shorter version of the Wechsler Adult Intelligence Scale-Fourth edition (WAIS-IV), Rey–Kim Memory Test, and Kims Frontal-Executive Neuropsychological Test.

1. **Jeonbuk National University Hospital (JBNU)**

The Structured Clinical Interview for DSM-IV (SCID-IV) was adopted for the diagnosis of schizophrenia. The psychiatric symptoms of patients with schizophrenia were assessed using the PANSS by trained psychiatrists in the week preceding the MRI scan. The exclusion criteria were as follows: 1) alcohol or drug dependency within the past six months; 2) intellectual disabilities; 3) current or historical neurological illness; 4) serious internal or external illness; and 5) positive urine pregnancy test. The control participants were age- and sex-matched with patients with schizophrenia and evaluated using the screening module of the SCID-IV, non-patient edition. The control participants did not have any previous or current psychiatric or neurological disorders or significant medical conditions.

1. **Center of Biomedical Research Excellence (COBRE)**

Multimodal neuroimaging data were obtained from patients with schizophrenia and age-matched controls and were released through the collaborative informatics and neuroimaging suite of the Mind Research Network. The SCID-IV was used to diagnose schizophrenia. The control participants had no personal or family history of major psychiatric disorders. Among the eligible participants, those with an IQ < 70, history of neurological disorders, severe head trauma, substance abuse or dependence within the last 12 months, and MRI contraindications were excluded.

1. **Neuromorphometry by Computer Algorithm Chicago (NMorphCH)**

A longitudinal study with a two-year follow-up was conducted at Northwestern University. It included clinical, cognitive, and neuroimaging data from patients with schizophrenia and healthy controls. The neuroimaging data included T1-weighted, T2-weighted, and diffusion-weighted images; resting-state functional MRI (fMRI); and an n-back task-based fMRI. The clinical symptoms of patients with schizophrenia were assessed using the Scale for the Assessment of Positive Symptoms (SAPS) and Scale for the Assessment of Negative Symptoms (SANS). Neurocognitive information on working memory, episodic memory, and executive function of the participants was included in the dataset.

1. **MIND Clinical Imaging Consortium (MCIC)**

This cross-sectional, multisite study was conducted to identify the neuroimaging biomarkers of schizophrenia. The MCIC dataset contains structural, functional, and diffusion-weighted MR images of patients with schizophrenia and age- and sex-matched controls, along with comprehensive clinical data. Patients with schizophrenia and a broad range of illness durations, from early psychosis to chronic illness, were included.

1. **University of California Los Angeles Consortium for Neuropsychiatric Phenomics LA5c Study (UCLA)**

This publicly available dataset includes neuroimaging data of healthy controls and patients with schizophrenia, bipolar disorder, or attention-deficit hyperactivity disorder, along with their neuropsychological and neurocognitive data. The primary goal of this project was to understand the dimensional structure of memory and cognitive control functions in non-patient and patient samples. We selectively retrieved clinical and neuroimaging data of patients with schizophrenia and healthy individuals from the entire dataset.

**Supplementary Table 1.** Scanners and T1-weighted MRI parameters

| Dataset | Scanners | Protocol parameters |
| --- | --- | --- |
| AMC 1 | 3T Philips Achieva | T1-weighted structural images were acquired with an 8-channel SENSE head-coil (TE = 4.6 ms, TR = 9.0 ms, voxel size = 1.0 × 1.0 × 1.0 mm, FOV = 240 × 240 × 170 mm, flip angle = 8°) |
| AMC 2 | 3T Philips Ingenia | TE = 4.6 ms, TR = 9.9 ms, voxel size = 1.0 × 1.0 × 1.0 mm, FOV = 240 × 240 × 170 mm, flip angle = 8° |
| AMC 3 | 3T Philips Ingenia CX | Sagittal T1-weighted structural images were acquired with a 32-channel dStream head coil and 3D, FFE sequence (TE = 2.9 ms, TR = 6.5 ms, flip angle = 9°, FOV (RL, AP, FH) = 211 × 256 × 256 mm, voxel size = 1.0 × 1.0 × 1.0 mm, 211 slices, slice thickness = 1 mm). |
| JBNU | 3T Siemens Verio | Axial T1-weighted spin-echo images were acquired (TR = 1 900 ms, TE = 2.5 ms, slice thickness = 1.0 mm, flip angle = 9°, FOV = 240 mm, and image matrix= 256 × 246 mm). |
| COBRE | 3T Siemens Trio | Coronal T1-weighted structural images were acquired with a 12-channel head-coil and a five-echo MPRAGE sequence (TE = 1.64, 3.5, 5.36, 7.22, and 9.08 ms; TR = 2.53 s; TI = 1.2 s, flip angle = 7°; number of excitations = 1; slice thickness = 1 mm; field of view = 256 mm; resolution = 256 × 256) |
| NMorphCH | NA | Coronal T1-weighted structural images were acquired with a 32-channel head coil and an MPRAGE sequence (TR = 2400 ms, TE = 3.16 ms, flip = 8°, 256 × 256 matrix, 176 slices, slice thickness = 1 mm) |
| MCIC | 3T Siemens Trio | Coronal T1-weighted images were acquired with an 8-channel head coil and a gradient echo sequence (TR = 12 ms; TE = 3.79 ms; flip angle = 20°; bandwidth = 181; voxel size = 0.625 × 0.625 mm; slice thickness = 1.5 mm; matrix = 256 × 256; number of slices = 128) |
| UCLA | 3T Siemens Trio | T1-weighted high-resolution anatomical (MPRAGE) images were acquired (slice thickness = 1 mm; 176 slices; TR = 1900 ms; TE = 2.26 ms; matrix = 256 × 256; FOV = 250 × 250 mm) |

AMC, Asan Medical Center; JBNU, Jeonbuk National University Hospital; COBRE, Center of Biomedical Research Excellence; NMorphCH, Neuromorphometry by Computer Algorithm Chicago; MCIC, MIND Clinical Imaging Consortium; UCLA, University of California Los Angeles Consortium for Neuropsychiatric Phenomics LA5c Study; TE, echo time; TI, inversion time; TR, repetition time; FOV, field of view; FFE, fast-field echo; MPRAGE, magnetization prepared rapid gradient echo; NA, not available.

**Outlier detection and ComBat harmonization**

To remove outliers from downstream analyses, we used the total number of defect holes in the cortical surface prior to fixing, as generated by the FreeSurfer automated pipeline. The Euler number, calculated based on the number of defect holes, is recognized as an objective quality control metric in structural MRI analysis (Rosen et al. 2018). For each cohort, we estimated the number of defect holes per subject and identified outliers, accounting for variations in scanners and acquisition parameters across cohorts. Subjects with values exceeding the threshold of 1.5 times the interquartile range (IQR) above the third quartile were identified as outliers (Supplementary Figure 1). In addition, all images were subjected to thorough visual inspection, and only those deemed of sufficient quality were retained for downstream analyses.


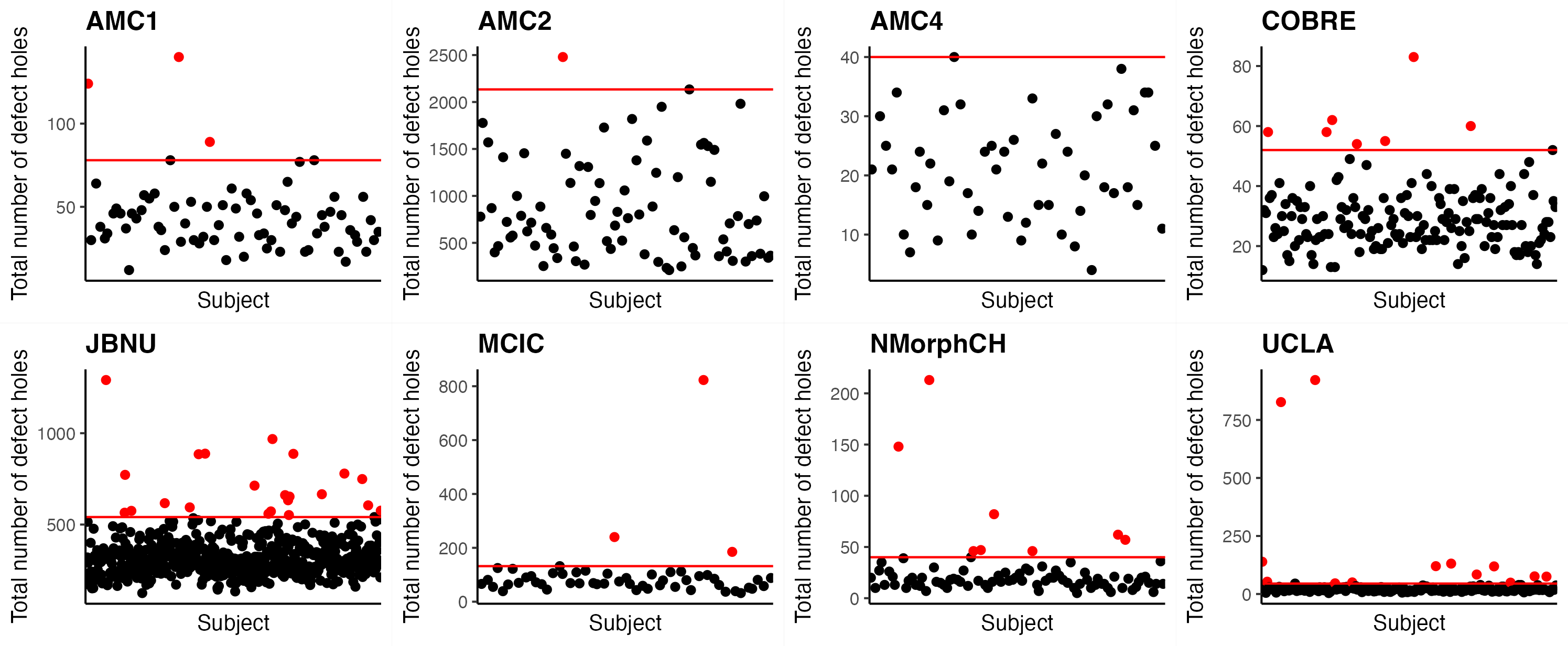


**Supplementary Figure 1. Distribution of the total number of defect holes.** Outliers were identified based on the threshold of 1.5 × IQR above the third quartile and are indicated as red dots. Subjects with numbers exceeding the threshold were excluded from subsequent analyses after additional visual inspection. IQR, interquartile range; AMC, Asan Medical Center; JBNU, Jeonbuk National University Hospital; COBRE, Center of Biomedical Research Excellence; NMorphCH, Neuromorphometry by Computer Algorithm Chicago; MCIC, MIND Clinical Imaging Consortium; UCLA, University of California Los Angeles Consortium for Neuropsychiatric Phenomics LA5c Study.

We performed ComBat harmonization to adjust for variability arising from the use of multiple neuroimaging datasets, incorporating age, sex, and diagnosis as covariates. Supplementary Figures 2–4 present the validation of the harmonization procedure at the connectivity, global, and nodal levels. In Supplementary Figure 2, principal component analysis was conducted to extract two principal components, and structural connectivity values from the Morphometric INverse Divergence (MIND) network were projected onto this two-dimensional space. Prior to harmonization, the connectivity profiles showed clear clustering by cohort, indicating pronounced site-specific effects. After harmonization, the data points were more uniformly distributed across cohorts, demonstrating effective attenuation of cohort-related variance in connectivity values. Consistent with these findings, Supplementary Figure 3 shows corresponding reductions in site-specific effects in global network metrics, while Supplementary Figure 4 demonstrates similar attenuation at the nodal level, supporting the effectiveness of ComBat harmonization across multiple scales of network organization.

**
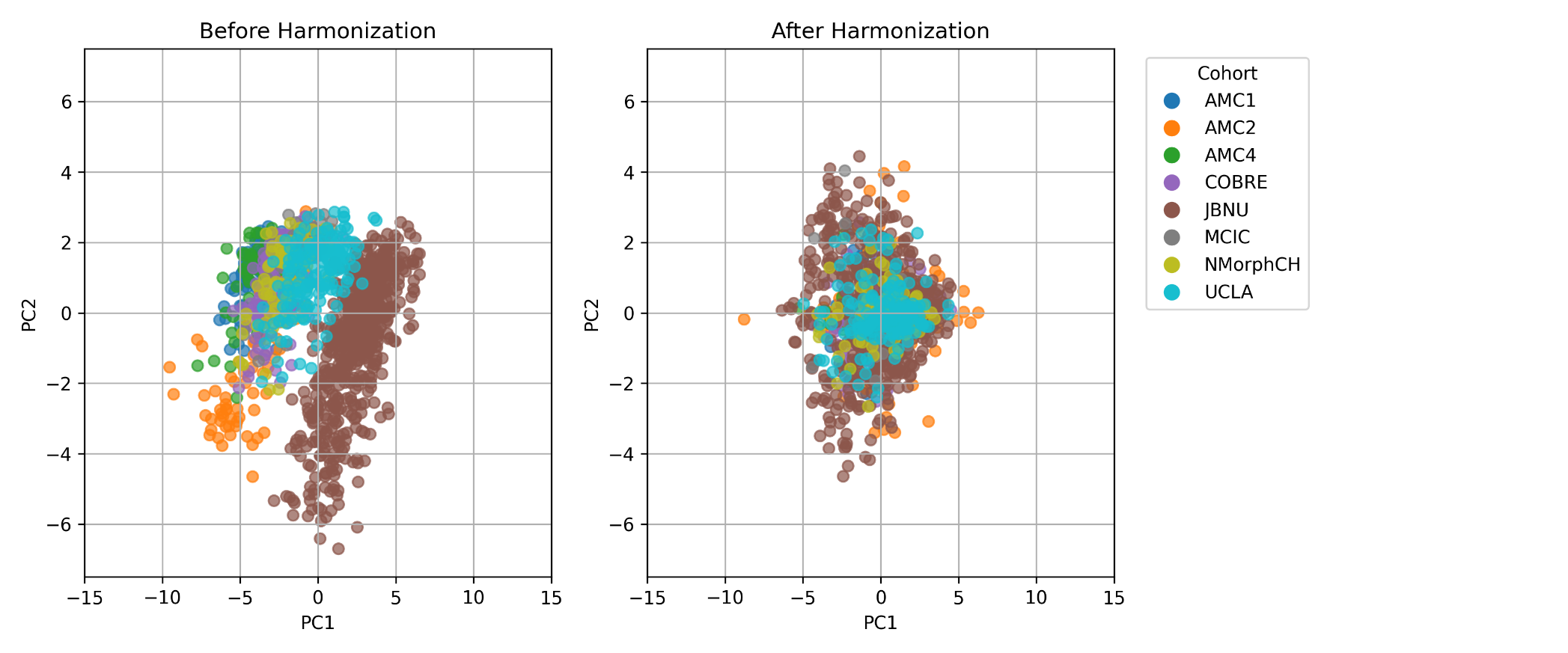
**

**Supplementary Figure 2. Principal component analysis (PCA) of MIND network structural connectivity values before and after ComBat harmonization.** Each point represents an individual subject, colored by cohort. MIND, Morphometric INverse Divergence; AMC, Asan Medical Center; JBNU, Jeonbuk National University Hospital; COBRE, Center of Biomedical Research Excellence; NMorphCH, Neuromorphometry by Computer Algorithm Chicago; MCIC, MIND Clinical Imaging Consortium; UCLA, University of California Los Angeles Consortium for Neuropsychiatric Phenomics LA5c Study.

| 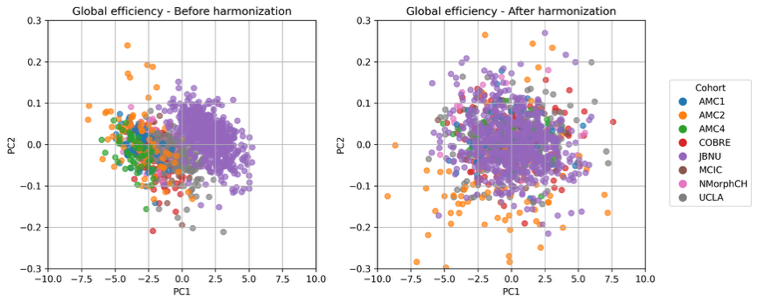 |
| --- |
| 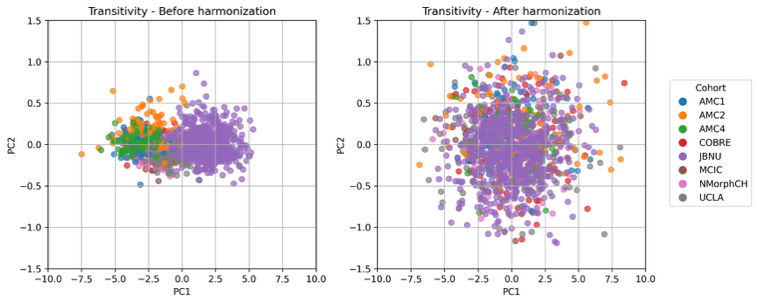 |
| 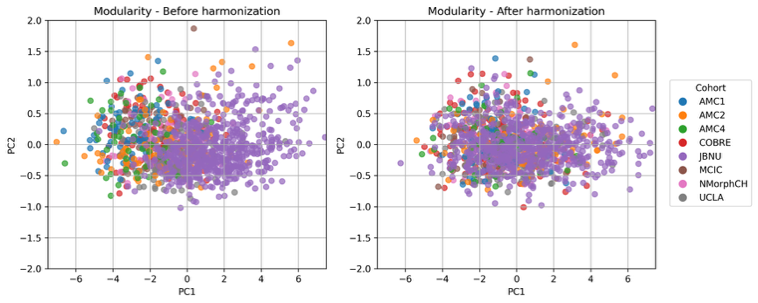 |
| 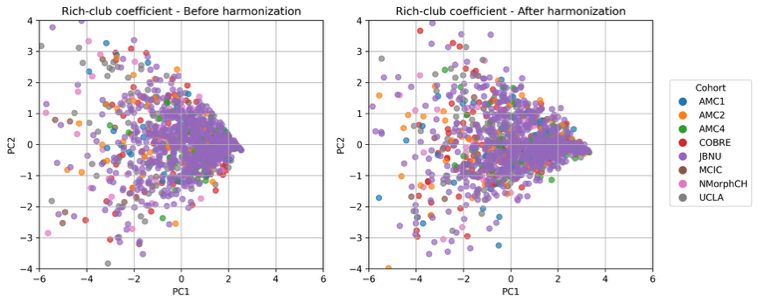 |
| 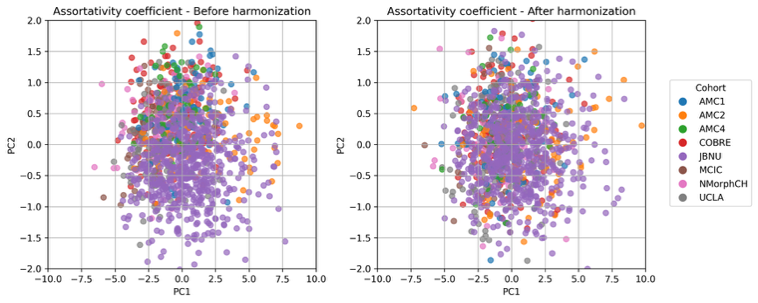 |

**Supplementary Figure 3. PCA of global network metrics before and after ComBat harmonization.** Each point represents an individual subject, colored by cohort. MIND, Morphometric INverse Divergence; AMC, Asan Medical Center; JBNU, Jeonbuk National University Hospital; COBRE, Center of Biomedical Research Excellence; NMorphCH, Neuromorphometry by Computer Algorithm Chicago; MCIC, MIND Clinical Imaging Consortium; UCLA, University of California Los Angeles Consortium for Neuropsychiatric Phenomics LA5c Study.

| 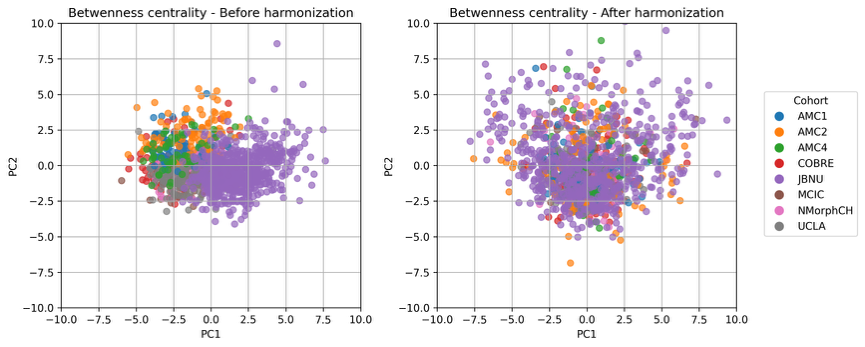 | 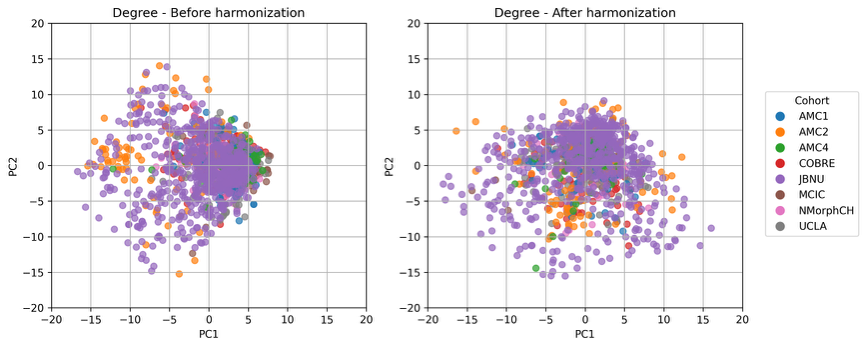 |
| --- | --- |
| 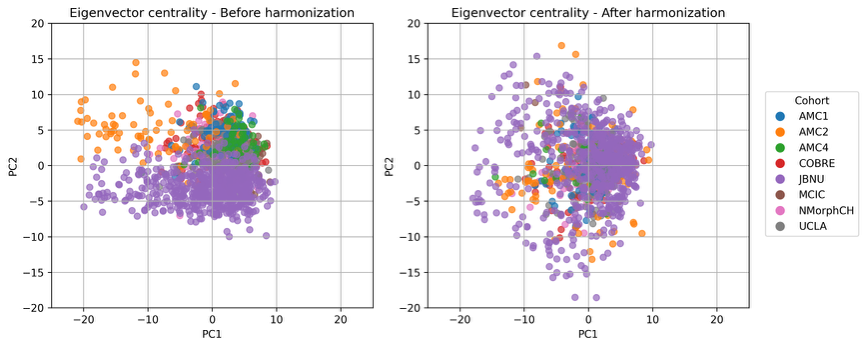 | 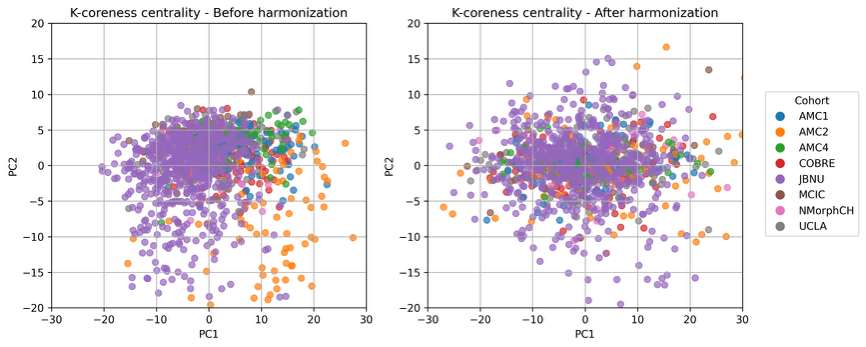 |
| 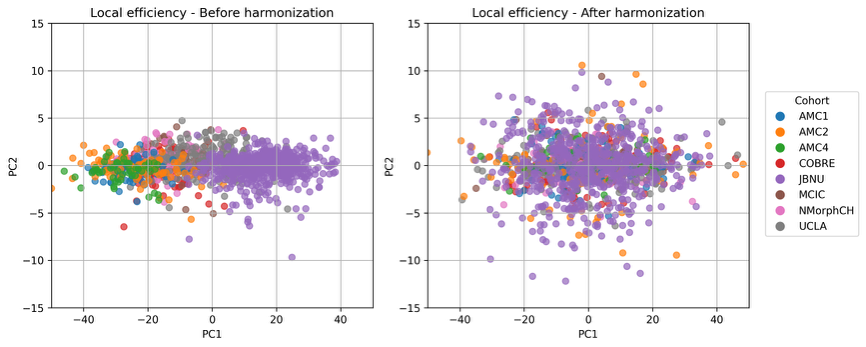 | 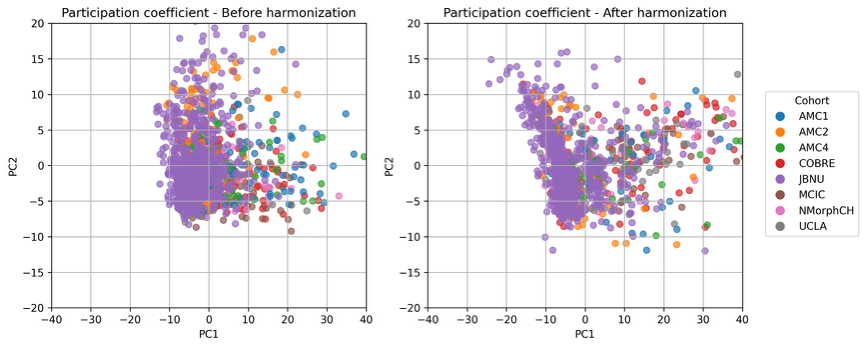 |

**Supplementary Figure 4. PCA of nodal network metrics before and after ComBat harmonization.** Each point represents an individual subject, colored by cohort. MIND, Morphometric INverse Divergence; AMC, Asan Medical Center; JBNU, Jeonbuk National University Hospital; COBRE, Center of Biomedical Research Excellence; NMorphCH, Neuromorphometry by Computer Algorithm Chicago; MCIC, MIND Clinical Imaging Consortium; UCLA, University of California Los Angeles Consortium for Neuropsychiatric Phenomics LA5c Study.

**Selection of optimal sparsity range for MIND network**

We assessed the small-worldness of the MIND network using proportional thresholding and network analysis implemented with the networkx library in Python. For each subject, structural connectivity matrices were thresholded across sparsity levels ranging from 0.10 to 0.50 in 0.05 increments. At each threshold, binarized and undirected graphs were constructed, and small-worldness was computed using a previously validated approach (Humphries and Gurney 2008). For each cohort, the mean and standard deviation of the small-worldness index (σ) were calculated across subjects at each threshold (Supplementary Figure 5). σ values greater than 1.2 were considered indicative of a meaningful small-world topology. Additionally, we examined the number of participants with valid small-worldness estimates at each sparsity level and identified the 0.25–0.50 range as robust. This range was therefore adopted for subsequent calculation of network metrics.


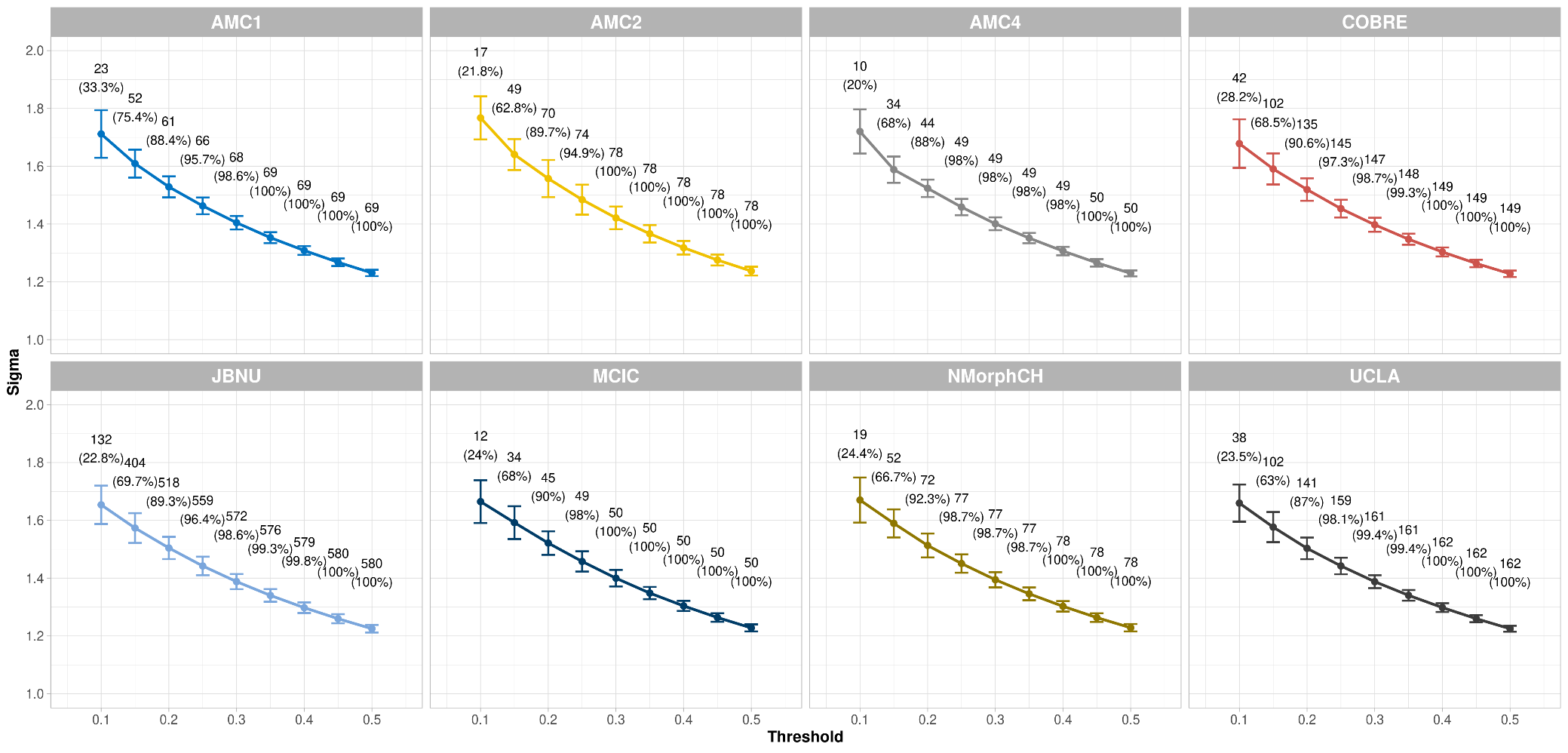


**Supplementary Figure 5. Small-worldness of MIND network across sparsity thresholds.** Each line shows the cohort-wise mean σ with error bars indicating the standard deviation. MIND, Morphometric INverse Divergence; AMC, Asan Medical Center; JBNU, Jeonbuk National University Hospital; COBRE, Center of Biomedical Research Excellence; NMorphCH, Neuromorphometry by Computer Algorithm Chicago; MCIC, MIND Clinical Imaging Consortium; UCLA, University of California Los Angeles Consortium for Neuropsychiatric Phenomics LA5c Study.

**Calculation of global and nodal network metrics**

We evaluated both global and nodal properties of the MIND networks using the Brain Connectivity Toolbox (BCT) (Rubinov and Sporns 2010). Network metrics were computed on binarized or weighted graphs depending on the metric, with proportional thresholding applied at sparsity levels ranging from 0.25 to 0.50 in 0.05 increments. Global metrics were computed using the following BCT functions:

- Global efficiency (efficiency_bin): Reflects the average inverse shortest path length, indicating parallel information transfer capacity.
- Transitivity (transitivity_bu): Represents the proportion of triangles relative to connected triplets, serving as an index of global clustering.
- Modularity (Q) (modularity_und): Quantifies the degree to which a network decomposes into communities with dense intra-module and sparse inter-module connections.
- Rich-club coefficient (maximum) (rich_club_bu): Captures the density of connections among high-degree nodes; the maximum value across k-levels was used.
- Assortativity coefficient (assortativity_bin): Assesses the tendency of nodes to connect with others of similar (or dissimilar) degree.

Nodal metrics included:

- Degree (degrees_und): The number of connections per node.
- Local efficiency (efficiency_wei): Reflects the communication efficiency among the neighbors of a node when the node is removed.
- Participation coefficient (participation_coef): Indicates how evenly the connections of a node are distributed across modules.
- Betweenness centrality (betweenness_wei): Measures how frequently a node lies on the shortest paths between other nodes.
- Eigenvector centrality (eigenvector_centrality_und): Reflects the importance of a node based on its connections to other central nodes.
- K-coreness centrality (kcoreness_centrality_bu): Indicates the maximal core a node belongs to, reflecting its embeddedness within the network.

Network metrics were calculated at each threshold independently. For statistical analysis, we computed the average of valid metric values across thresholds (0.25–0.50) for each subject, minimizing the influence of threshold-specific instability and missing values.

**Supplementary Table 2. Group comparisons of global network metrics**

|  | HC | | SCZ | |  |  |  |
| --- | --- | --- | --- | --- | --- | --- | --- |
| Network metric | Mean | SD | Mean | SD | t-stat | FDR p | partial R² |
| Global efficiency, 10^-3^ | 128.268 | 4.954 | 128.493 | 4.966 | 1.097 | 0.273 | 0.001 |
| Transitivity, 10^-3^ | 88.059 | 3.869 | 88.242 | 3.857 | 1.182 | 0.273 | 0.001 |
| Modularity, 10^-3^ | 162.335 | 11.518 | 163.412 | 12.369 | 1.408 | 0.273 | 0.002 |
| Rich-club coefficient, 10^-4^ | 9999.191 | 43.9 | 9999.268 | 39.9 | 3.203 | 0.007 | 0.008 |
| Assortativity coefficient, 10^-3^ | 101.509 | 19.121 | 102.920 | 16.277 | 1.214 | 0.273 | 0.001 |

HC, healthy controls; SCZ, schizophrenia; SD, standard deviation; t-stat, t-statistic; FDR p, false discovery rate-corrected p value.


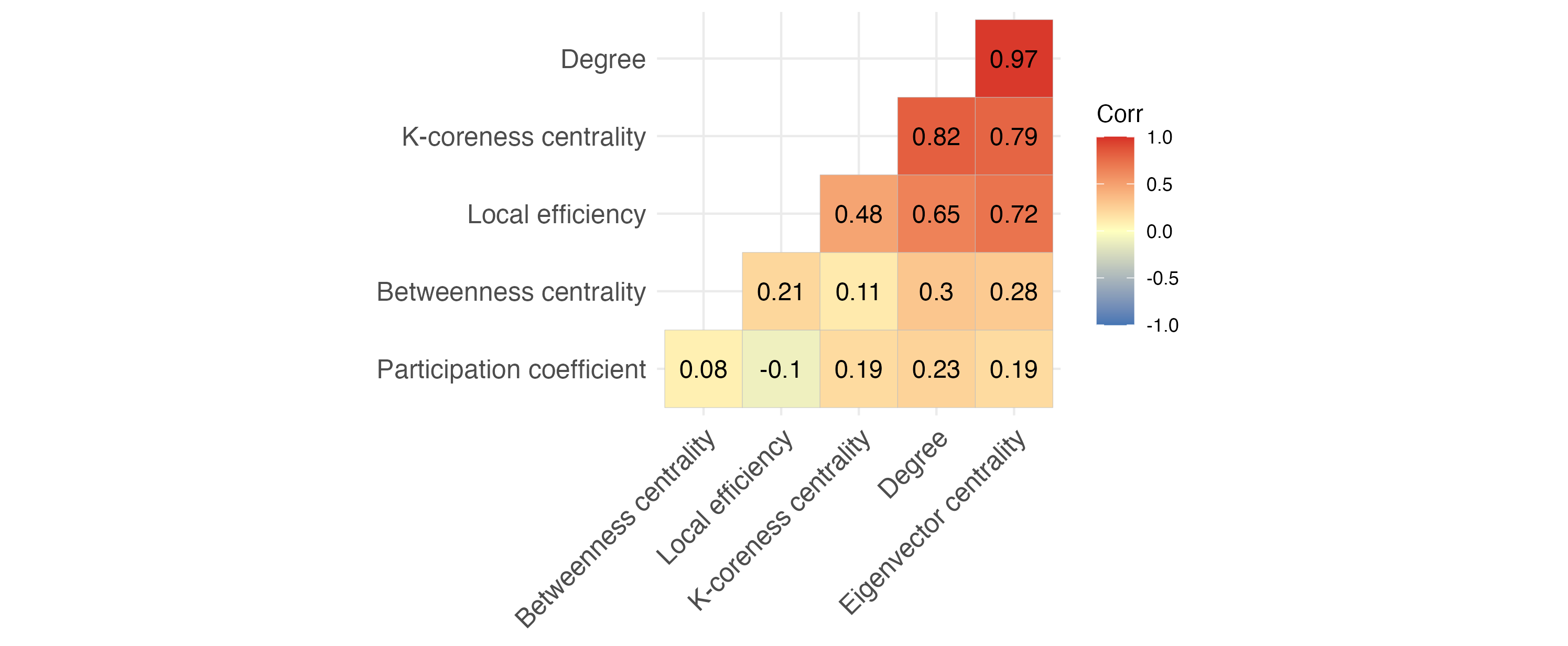


**Supplementary Figure 6. Pearson correlation matrix of case-control t-statistics across nodal network metrics.** Warmer colors indicate stronger positive correlations, and cooler colors indicate negative correlations. Strong correlations were observed between degree, k-coreness centrality, and eigenvector centrality, while betweenness centrality and participation coefficient showed relatively weak correlations with other metrics.

**Supplementary Table 3. Group comparisons for nodal network metrics across the Yeo functional networks**

|  | Visual | | Somatomotor | | Dorsal attention | | Salience/Ventral attention | | Limbic | | Control | | Default | |  | |
| --- | --- | --- | --- | --- | --- | --- | --- | --- | --- | --- | --- | --- | --- | --- | --- | --- |
| Network metric | Mean | SD | Mean | SD | Mean | SD | Mean | SD | Mean | SD | Mean | SD | Mean | SD | F | p-value |
| Betweenness centrality | 0.178 | 1.175 | 0.19 | 1.165 | 0.3 | 1.214 | 0.39 | 1.133 | -0.008 | 1.111 | 0.465 | 1.327 | 0.089 | 1.323 | 0.870 | 0.517 |
| Degree | 0.854 | 1.612 | -0.105 | 1.703 | 0.525 | 1.101 | -0.362 | 1.26 | 0.012 | 1.377 | 0.356 | 1.044 | -0.296 | 1.069 | 6.841 | <0.001 |
| Eigenvector centrality | 1.052 | 1.714 | -0.173 | 1.743 | 0.466 | 1.133 | -0.584 | 1.337 | 0.186 | 1.37 | 0.492 | 1.127 | -0.3 | 1.086 | 9.575 | <0.001 |
| K-coreness centrality | 0.599 | 1.703 | -0.425 | 1.505 | -0.123 | 1.272 | -0.968 | 1.036 | -0.566 | 1.461 | -0.57 | 1.154 | -0.939 | 1.041 | 10.241 | <0.001 |
| Local efficiency | 1.534 | 1.184 | 0.494 | 1.208 | 0.872 | 0.895 | 0.643 | 1.044 | 1.272 | 1.199 | 1.391 | 0.919 | 0.826 | 0.948 | 8.282 | <0.001 |
| Participation coefficient | -1.434 | 1.003 | -1.65 | 1.184 | -0.832 | 1.28 | -1.161 | 1.306 | -1.617 | 1.406 | -1.386 | 1.068 | -1.361 | 1.096 | 2.845 | 0.010 |

SD, standard deviation.


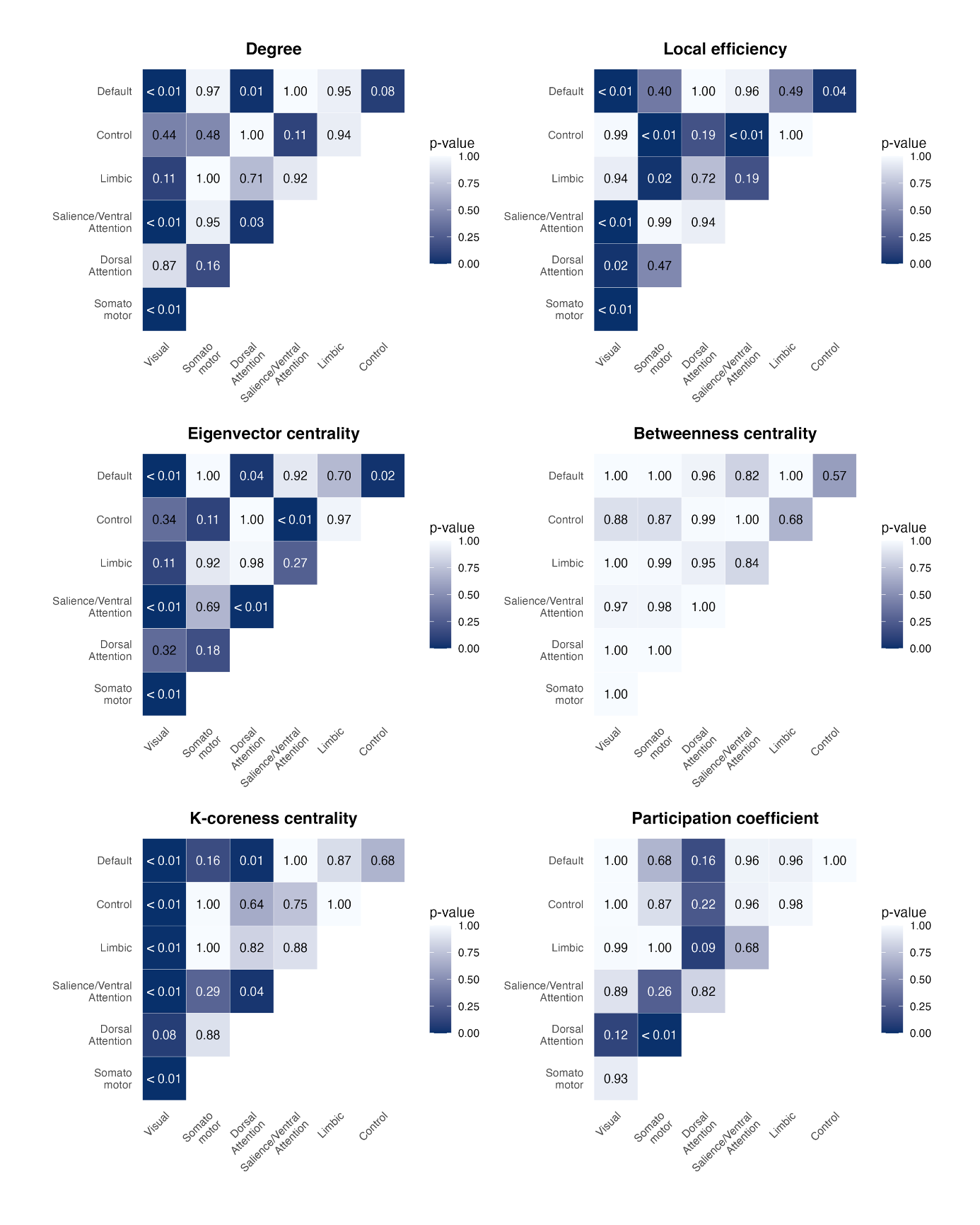


**Supplementary Figure 7. Pairwise post-hoc comparisons of case–control t-statistics across Yeo’s seven functional networks (p-values from Tukey’s HSD test).** Darker colors indicate lower p-values. HSD, honestly significant difference.


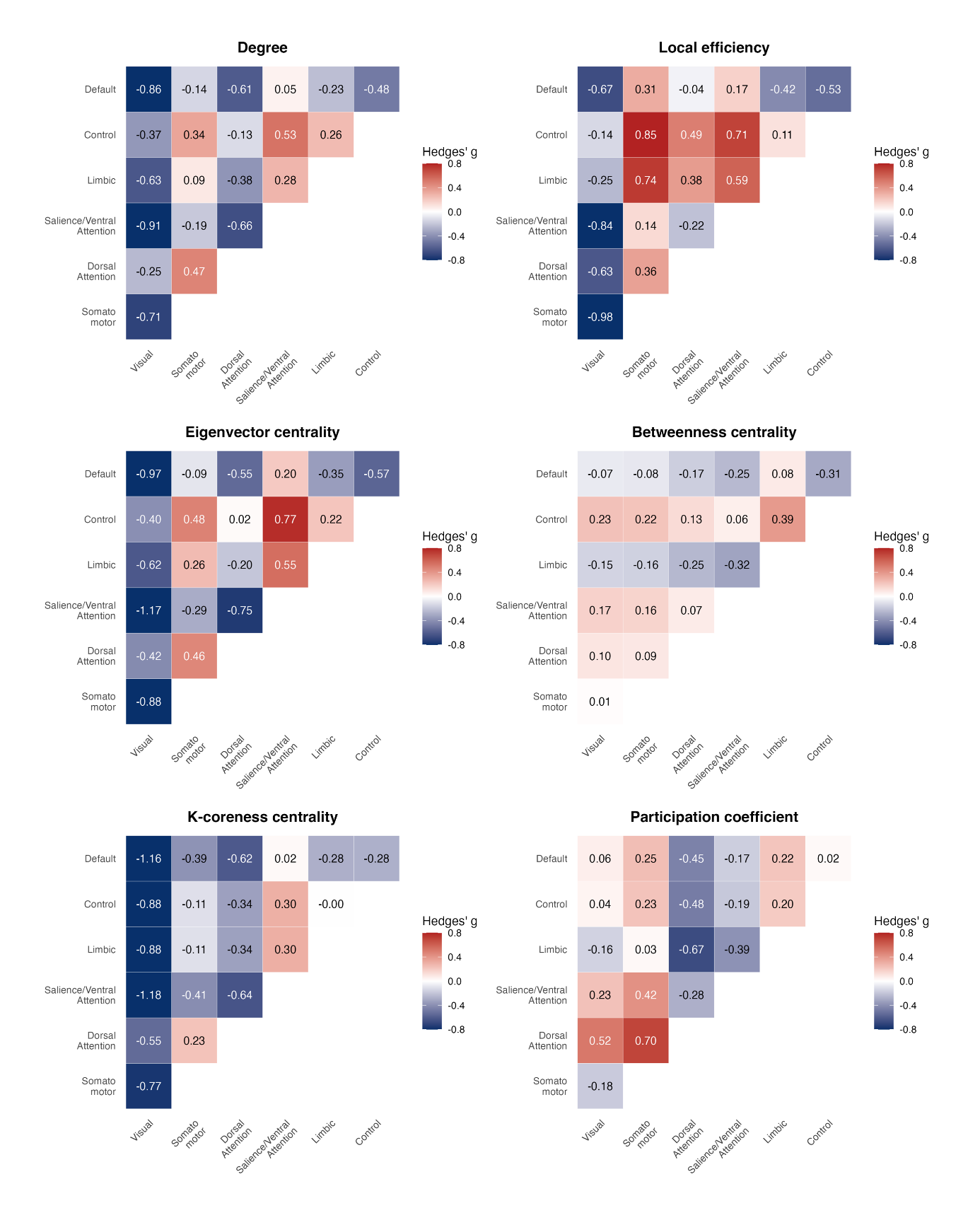


**Supplementary Figure 8. Pairwise post-hoc comparisons of case–control t-statistics across Yeo’s seven functional networks (effect sizes expressed as Hedges’ g).** Darker red and blue colors indicate higher and lower effect sizes, respectively.

**Supplementary Table 4. Clinical associations of the nodal network metrics**

| Region | Network metric | Clinical variable | rho | FDR p |
| --- | --- | --- | --- | --- |
| 7Networks_RH_Vis_20 | Local efficiency | PANSS negative | 0.141 | 0.012 |
| 7Networks_LH_Vis_10 | Participation coefficient | PANSS negative | 0.113 | 0.042 |
| 7Networks_LH_Vis_13 | Participation coefficient | PANSS negative | 0.169 | 0.001 |
| 7Networks_LH_SomMot_26 | Participation coefficient | PANSS negative | 0.126 | 0.021 |
| 7Networks_LH_SomMot_32 | Participation coefficient | PANSS negative | 0.112 | 0.042 |
| 7Networks_LH_SomMot_37 | Participation coefficient | PANSS negative | 0.11 | 0.043 |
| 7Networks_LH_Default_pCunPCC_4 | Participation coefficient | PANSS negative | 0.134 | 0.014 |
| 7Networks_RH_SomMot_39 | Participation coefficient | PANSS negative | 0.112 | 0.042 |
| 7Networks_RH_SomMot_40 | Participation coefficient | PANSS negative | 0.138 | 0.012 |
| 7Networks_RH_Limbic_OFC_4 | Participation coefficient | PANSS negative | 0.117 | 0.041 |
| 7Networks_LH_SomMot_5 | Degree | FSIQ | 0.207 | 0.03 |
| 7Networks_LH_SomMot_17 | Participation coefficient | FSIQ | 0.215 | 0.03 |
| 7Networks_LH_Limbic_OFC_4 | Participation coefficient | FSIQ | 0.209 | 0.03 |
| 7Networks_LH_Default_PFC_8 | Participation coefficient | FSIQ | 0.201 | 0.03 |
| 7Networks_RH_SomMot_26 | Participation coefficient | FSIQ | 0.202 | 0.03 |
| 7Networks_RH_DorsAttn_Post_1 | Participation coefficient | FSIQ | 0.226 | 0.03 |
| 7Networks_RH_Vis_20 | Eigenvector centrality | FSIQ | -0.2 | 0.03 |
| 7Networks_RH_Vis_24 | Eigenvector centrality | FSIQ | -0.202 | 0.03 |
| 7Networks_LH_SomMot_5 | K-coreness centrality | FSIQ | 0.214 | 0.03 |
| 7Networks_RH_Vis_24 | K-coreness centrality | FSIQ | -0.21 | 0.03 |
| 7Networks_RH_Vis_12 | Local efficiency | Illness duration | 0.206 | <0.001 |
| 7Networks_RH_Vis_20 | Local efficiency | Illness duration | 0.189 | <0.001 |
| 7Networks_RH_Limbic_TempPole_7 | Local efficiency | Illness duration | 0.149 | 0.007 |
| 7Networks_RH_Limbic_OFC_4 | Participation coefficient | Illness duration | 0.143 | 0.008 |
| 7Networks_LH_SalVentAttn_Med_2 | Eigenvector centrality | Illness duration | -0.125 | 0.035 |

FSIQ, full-scale intelligence quotient; PANSS, Positive and Negative Syndrome Scale; LH, left hemisphere; RH, right hemisphere; Vis, visual network; SomMot, somatomotor network; DorsAttn, dorsal attention network; SalVentAttn, salience/ventral attention network; Limbic, limbic network; Default, default mode network; PFC, prefrontal cortex; pCunPCC, precuneus/posterior cingulate cortex; OFC, orbitofrontal cortex; FDR p, false discovery rate-corrected p value.

**
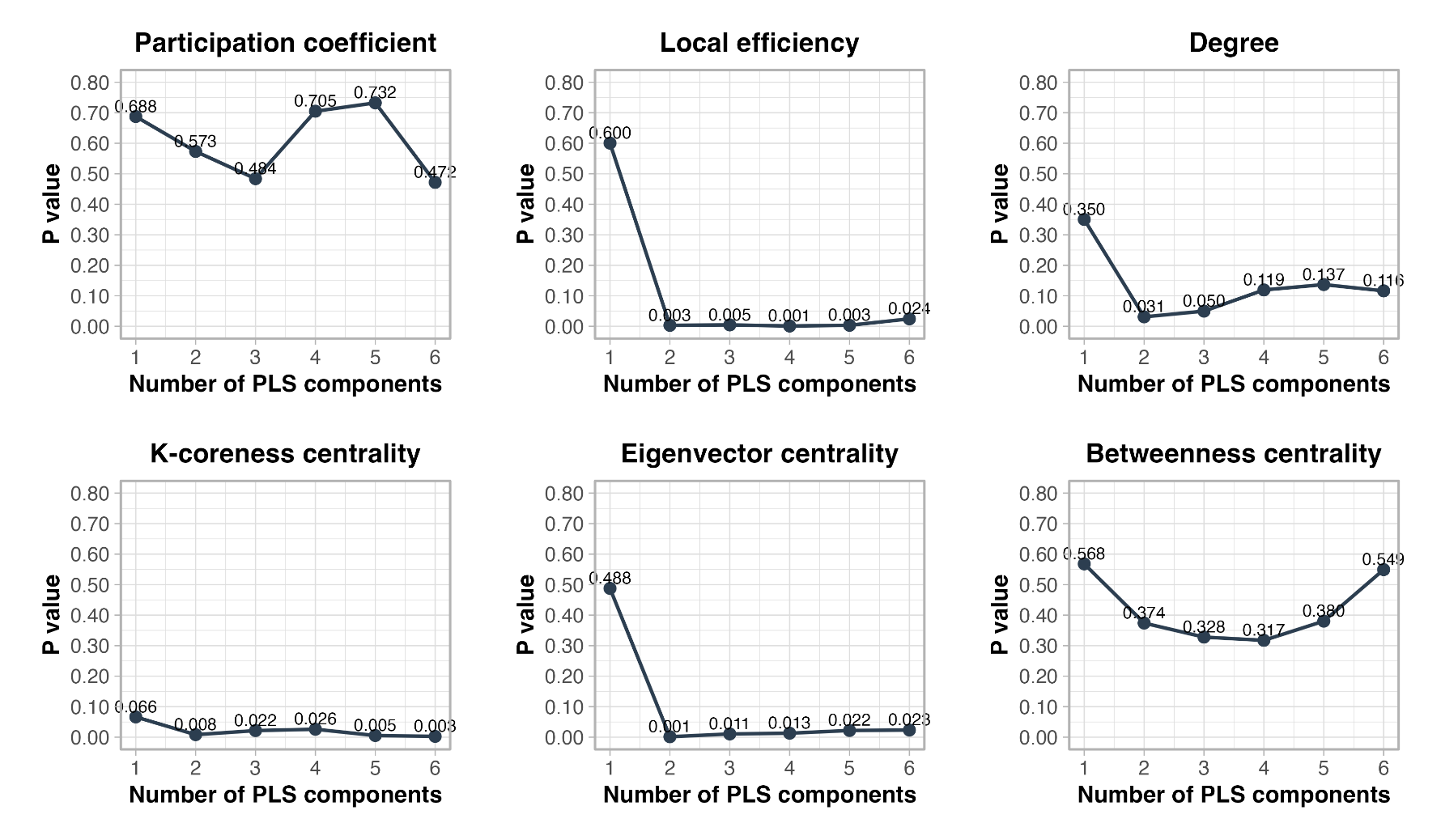
**

**Supplementary Figure 9. Permutation-based p-values according to the number of partial least squares (PLS) components.** Permutation-derived p-values for the first six components of the PLS regression linking nodal network alterations to cortical gene expression are shown. Statistical significance was assessed using 5,000 permutations of the response variable, which consisted of regional case–control t-values from each nodal network metric.


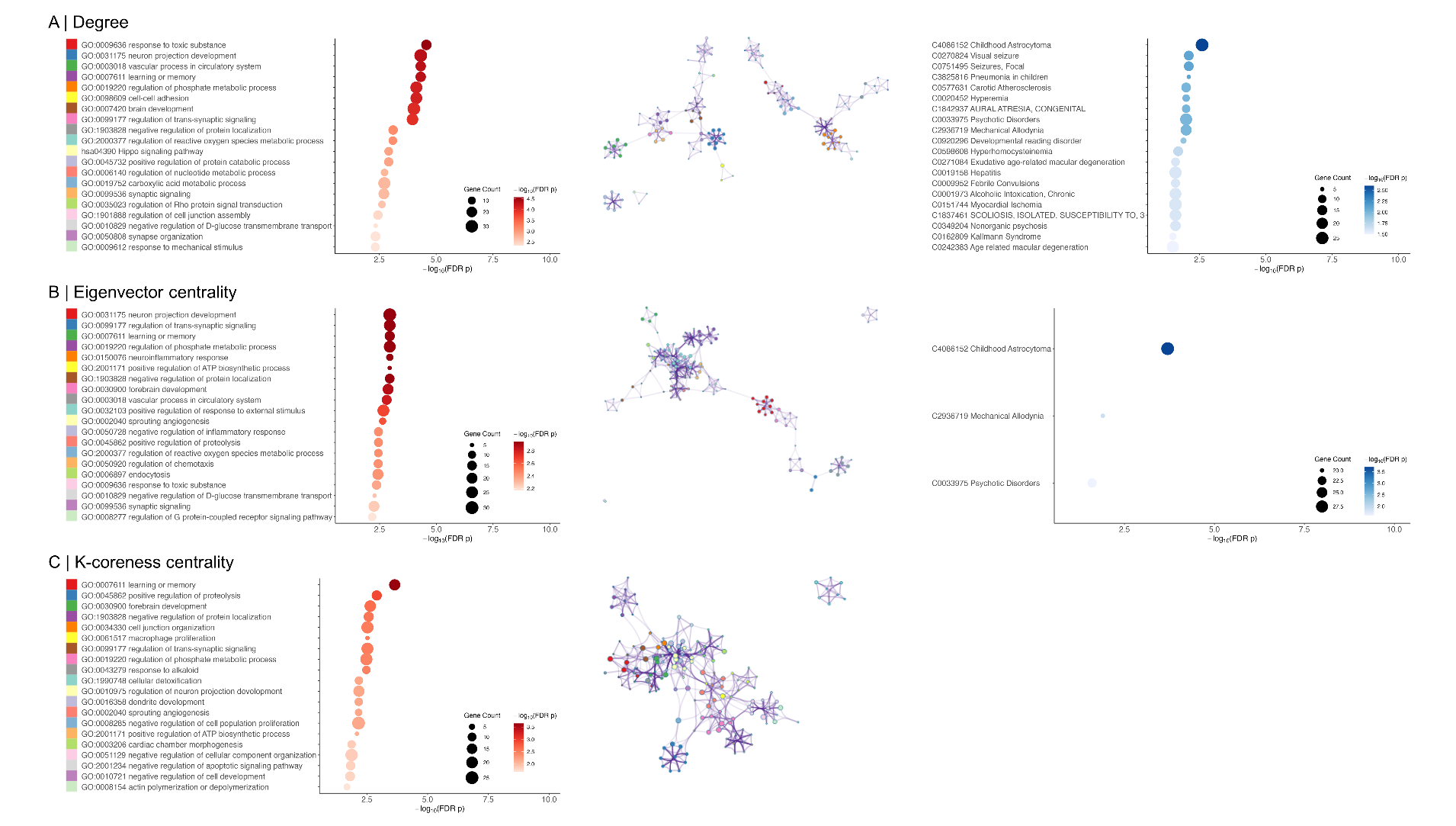


**Supplementary Figure 10.** Functional and disease enrichment of PLS2-positive gene sets associated with nodal network metrics. (A–C) Gene Ontology (GO) biological process, KEGG pathway, and disease enrichment results for PLS2-positive gene sets identified from degree (A), eigenvector centrality (B), and k-coreness centrality (C). Dot plots display significantly enriched terms (FDR < 0.05), with color intensity reflecting –log₁₀(p-value) and point size indicating gene count.

| **Degree** | **Eigenvector centrality** | **K-coreness centrality** |
| --- | --- | --- |
| 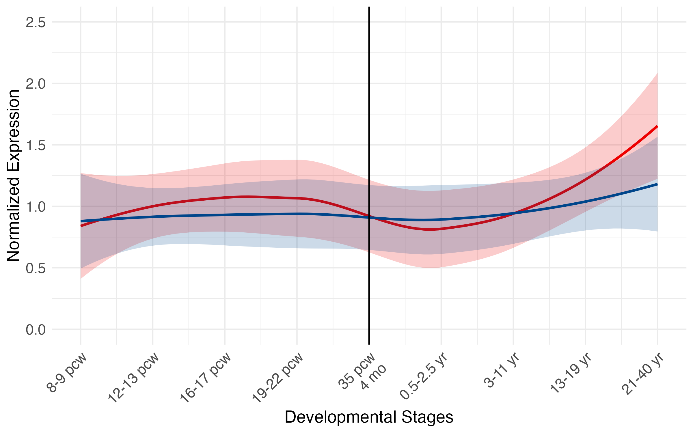 | 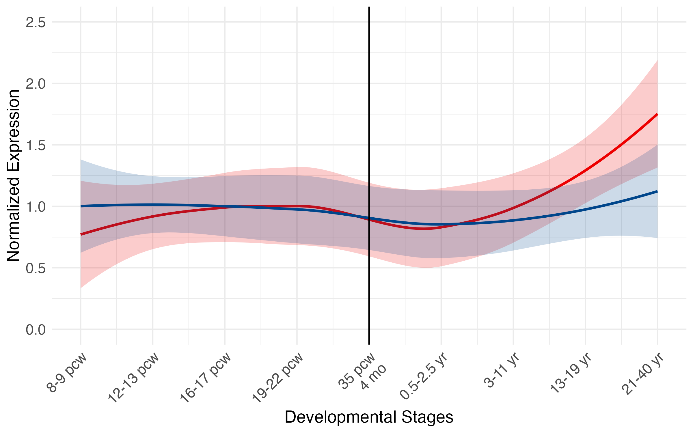 | 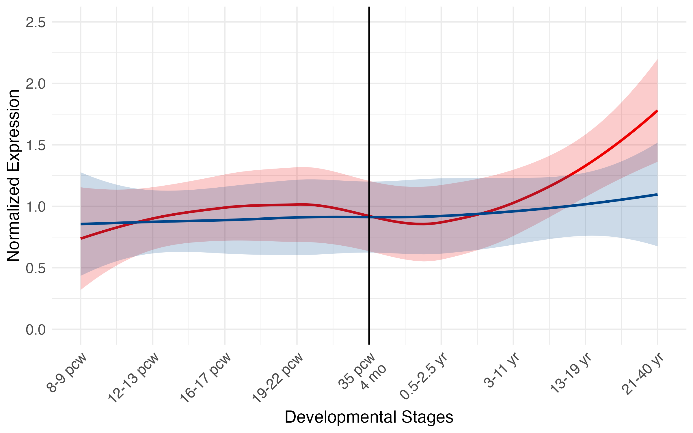 |

**Supplementary Figure 11**. **Developmental expression trajectories of PLS2-associated gene sets.** Normalized cortical expression levels of PLS2-positive (blue) and PLS2-negative (red) genes across nine developmental windows, derived from the PsychENCODE transcriptomic atlas. Expression values were log-transformed, centered across samples, and averaged across cortical regions. Shaded areas represent 95% confidence interval. The vertical line indicates the transition from prenatal to postnatal development (35 post-conceptional weeks/4 months). pcw, post-conceptional weeks; mo, months;

**Supplementary Table 5. Comparison between normalized expression levels of PLS2-positive and PLS2-negative gene sets across developmental windows**

| Developmental window | Degree | | Eigenvector centrality | | K-coreness  centrality | |
| --- | --- | --- | --- | --- | --- | --- |
|  | t | p | t | p | t | p |
| 8–9 pcw | -0.340 | 0.734 | -1.047 | 0.295 | -0.721 | 0.471 |
| 12–13 pcw | 1.528 | 0.127 | -0.766 | 0.444 | 1.301 | 0.194 |
| 16–17 pcw | 1.423 | 0.155 | 0.233 | 0.816 | 0.899 | 0.369 |
| 19–22 pcw | 1.654 | 0.099 | 0.587 | 0.558 | 1.596 | 0.111 |
| 0.5–2.5 yr | -1.559 | 0.120 | -0.714 | 0.476 | -1.592 | 0.112 |
| 3–11 yr | -0.431 | 0.667 | 0.443 | 0.658 | 0.592 | 0.554 |
| 13–19 yr | 1.941 | 0.053 | 3.353 | 0.001 | 3.047 | 0.002 |
| 21–40 yr | 5.416 | < 0.001 | 7.050 | < 0.001 | 7.607 | < 0.001 |

pcw, post-conception week; yr, years.

**Reference**

Humphries MD, Gurney K. 2008. Network 'small-world-ness': a quantitative method for determining canonical network equivalence. PLoS One. 3(4):e0002051.

Rosen AFG, Roalf DR, Ruparel K, Blake J, Seelaus K, Villa LP, Ciric R, Cook PA, Davatzikos C, Elliott MA et al. 2018. Quantitative assessment of structural image quality. Neuroimage. 169:407-418.

Rubinov M, Sporns O. 2010. Complex network measures of brain connectivity: Uses and interpretations. NeuroImage. 52(3):1059-1069.
